# Cooperative Domain Formation by Homologous Motifs in HOIL-1L and SHARPIN Plays A Crucial Role in LUBAC Stabilization

**DOI:** 10.1016/j.celrep.2018.03.112

**Published:** 2018-04-24

**Authors:** Hiroaki Fujita, Akira Tokunaga, Satoshi Shimizu, Amanda L. Whiting, Francisco Aguilar-Alonso, Kenji Takagi, Erik Walinda, Yoshiteru Sasaki, Taketo Shimokawa, Tsunehiro Mizushima, Izuru Ohki, Mariko Ariyoshi, Hidehito Tochio, Federico Bernal, Masahiro Shirakawa, Kazuhiro Iwai

**Affiliations:** 1 Department of Molecular and Cellular Physiology, Kyoto University School of Medicine, Kyoto 606-8501, Japan; 2 Department of Molecular Engineering, Kyoto University School of Engineering, Kyoto 615-8510, Japan; 3 Department of Anesthesia, Kyoto University Hospital, Kyoto 606-8507, Japan; 4 Laboratory of Protein Dynamics and Signaling, Center for Cancer Research, National Cancer Institute, Frederick, MD 21702, USA; 5 Department of Picobiology, University of Hyogo School of Life Science, Hyogo 678-1297, Japan; 6 Department of Biophysics, Kyoto University School of Science, Kyoto 606-8502, Japan; 7 These authors contributed equally; 8 Present address: Graduate School of Frontier Biosciences, Osaka University, Suita, Osaka 565-0871, Japan; 9 Lead Contact

## Abstract

The linear ubiquitin chain assembly complex (LUBAC) participates in inflammatory and oncogenic signaling by conjugating linear ubiquitin chains to target proteins. LUBAC consists of the catalytic HOIP subunit and two accessory subunits, HOIL-1L and SHARPIN. Interactions between the ubiquitin-associated (UBA) domains of HOIP and the ubiquitin-like (UBL) domains of two accessory subunits are involved in LUBAC stabilization, but the precise molecular mechanisms underlying the formation of stable trimeric LUBAC remain elusive. We solved the co-crystal structure of the binding regions of the trimeric LUBAC complex and found that LUBAC-tethering motifs (LTMs) located N terminally to the UBL domains of HOIL-1L and SHARPIN heterodimerize and fold into a single globular domain. This interaction is resistant to dissociation and plays a critical role in stabilizing trimeric LUBAC. Inhibition of LTM-mediated HOIL-1L/SHARPIN dimerization profoundly attenuated the function of LUBAC, suggesting LTM as a superior target of LUBAC destabilization for anticancer therapeutics.

## In Brief

Fujita et al. report a crystal structure of the trimeric LUBAC core and show that motifs in HOIL-1L and SHARPIN fold into a single domain critical for LUBAC stabilization. The authors also develop an inhibitor of this interaction that destabilizes LUBAC and kills cancer cells.

## Graphical Abstract

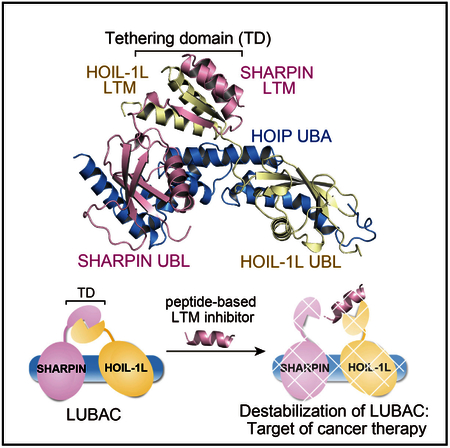

## INTRODUCTION

Protein ubiquitination modulates a wide range of biological functions (Hershko and Ciechanover, 1998; Husnjak and Dikic, 2012; Komander and Rape, 2012). Cells contain several types of ubiquitin chains, and the various chain types regulate their substrates in different manners. Chains can be generated by conjugation to the lysine residues of ubiquitin. In addition, linear ubiquitin chains linked via the N-terminal Met1 are generated by the linear ubiquitin chain assembly complex (LUBAC), which consists of the catalytic HOIP and the accessory HOIL-1L and SHARPIN subunits (Iwai et al., 2014; Kirisako et al., 2006). Both SHARPIN and HOIL-1L interact with HOIP via their ubiquitin-like (UBL) domains, and the two interactions are thought to sufficiently stabilize LUBAC (Gerlach et al., 2011; Ikeda et al., 2011; Kirisako et al., 2006; Tokunaga et al., 2011). LUBAC-catalyzed linear ubiquitination of the nuclear factor κB (NF-κB) essential modulator (NEMO), a key regulator of the IκB kinase (IKK) complex, induces oligomerization of the IKK complex via recognition of linear chains by the UBAN domain of another NEMO molecule, leading to activation of IKK by trans-autophosphorylation (Fujita et al., 2014; Kensche et al., 2012; Tokunaga et al., 2009; Zhang et al., 2014). In addition to NEMO, several proteins such as TNFR1, RIP1, RIP2, IRAK1, and MyD88, which are known to be involved in NF-κB activation, have been reported to be substrates of LUBAC (Emmerich et al., 2013; Fiil et al., 2013; Gerlach et al., 2011; Wertz et al., 2015), indicating crucial roles of LUBAC in NF-κB activation. LUBAC also suppresses formation of the cell death complex consisting of RIP1, FADD, and caspase-8 in a NF-κB-independent manner (Ashkenazi and Salvesen, 2014; Kumari et al., 2014; Lafont et al., 2017; Shimizu et al., 2016). Thus, it is now widely accepted that linear ubiquitin chains are involved in NF-κB signaling and regulation of cell death (Hrdinka and Gyrd-Hansen, 2017; Iwai et al., 2014; Peltzer et al., 2016). Mice lacking LUBAC ligase activity exhibit high rates of cell death indicated by embryonic lethality at embryonic day 10.5 (E10.5) (Emmerich et al., 2013; Peltzer et al., 2014; Sasaki et al., 2013; Shimizu et al., 2016). This evidence highlights the essential functionality of linear ubiquitination. Furthermore, mutant mice lacking SHARPIN develop severe autoinflammatory disease and immunodeficiency due to destabilization of the two remaining LUBAC subunits (Gerlach et al., 2011; Ikeda et al., 2011; Tokunaga et al., 2011). Therefore, it would be of great value to dissect the molecular mechanism underlying stabilization of the trimeric LUBAC complex.

In this study, we found that HOIL-1L is critical in stabilizing trimeric LUBAC. Like HOIP knockout (KO) mice, the newly generated HOIL-1L-null mice, which lack the UBL domain that serves as the binding site for HOIP, exhibited embryonic lethality as the result of LUBAC destabilization. To elucidate the molecular mechanisms underlying stabilization, we determined the crystal structure of the binding core regions of trimeric LUBAC. Surprisingly, the crystallographic analyses revealed that HOIL-1L and SHARPIN interact with each other via heterodimerization of newly identified LUBAC-tethering motifs (LTMs) in both proteins. Because the LTM dimer ultimately folds into a single globular domain, the interaction was resistant to dissociation; thus this interaction likely plays a predominant role in stabilization of trimeric LUBAC. Indeed, peptide-based inhibition of this interaction effectively destabilized LUBAC and suppressed the proliferation of B cell lymphoma cells, whose survival is dependent on LUBAC (Yang et al., 2014). In light of the involvement of LUBAC in the pathogenesis of B cell lymphomas and the resistance of cancers to immune checkpoint blockade therapy and cisplatin (MacKay et al., 2014; Manguso et al., 2017; Yang et al., 2014), our results sug gest that the abundance of LUBAC could be controlled by inhibiting the HOIL-1L/SHARPIN interaction.

## RESULTS

### Disruption of the HOIL-1L UBL Results in Embryonic Lethality at Midgestational Stage in Mice

**Figure 1.**
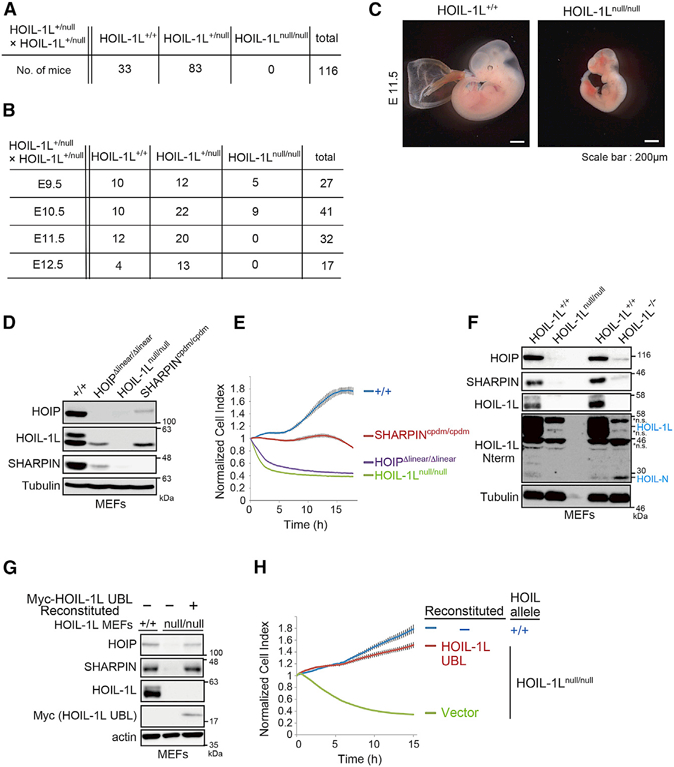
Disruption of HOIL-1L UBL Results in Embryonic Lethality (A) Number of offspring of each genotype resulting from crosses of HOIL-1L^+/null^” mice. (B) Numbers of embryos obtained at each embryonic stage (E9.5, 10.5, 11.5, and 12.5) from crosses of HOIL-1L^+/null^” mice. (C) Representative gross appearance of HOIL-1L-null and WT littermate on embryonic day 11.5 (E11.5). (D) Immunoblot analyses of lysates of MEFs from mice of the indicated genotypes. (E) Indicated MEFs were stimulated with TNF-α (10 ng/ml), and cell viability was continuously measured on a real-time cell analyzer (RTCA) (means ± SEM, n = 3). (F) Immunoblot analysis of lysates of MEFs from mice of the indicated genotypes. ns, non-specific band. (G) Immunoblot analysis of lysates of HOIL-1L-null MEFs reconstituted with HOIL-1L UBL. (H) Cell viability of the indicated MEFs was measured as described in Figure 1E (means ± SEM, n = 4). See also Figure S1.

In humans, HOIL-1L mutations cause two distinct sets of phenotypes: one is immunodeficiency, autoinflammation, and cardiomyopathy, and the other is polyglucosan body myopathy, including cardiomyopathy without immunological symptoms (Boisson et al., 2012; Nilsson et al., 2013). Our previously described HOIL-1L^–/–^ mice had a deleted C-terminal RING domain and exhibited polyglucosan body myopathy in old age (MacDuff et al., 2015). Because the N-terminal region of HOIL-1L, including the UBL that serves as the binding site for HOIP, is not thought to be expressed in patients with immunological symptoms (Boisson et al., 2012), we generated new HOIL-1L mutant mice with deletions in the N-terminal UBL. The mutations were introduced using two different CRISPR/Cas9 guide RNAs (Figures S1A and S1B). The *HOIL-1L*^*null/null*^ mice generated by both constructs exhibited embryonic lethality and died around E10.5, as observed in HOIP^–/–^ mice or mice lacking the HOIP ligase activity (*HOIP*^*Δlinear/Δlinear*^*)* (Figures 1A, 1B, S1C, and S1D) (Peltzer et al., 2014; Shimizu et al., 2016). HOIL-1L^*null/null*^ mice exhibited the same phenotypes as HOIP^–/–^ mice, including intracranial and/or thoracoabdominal hemorrhages; significantly higher levels of TUNEL-positive cells than in their wild-type (WT) littermates; and vascular defects in embryo (Figures 1C and S1E–S1G) (Peltzer et al., 2014). Also, HOIL-1L^*null/null*^ mouse embryonic fibroblasts (MEFs) were approximately as sensitive as HOIP^*Δlinear/Δlinear*^ MEFs to tumor necrosis factor alpha (TNF-α)-induced cell death (Figures 1D and 1E).

Immunoblotting confirmed that full-length HOIL-1L was not expressed in MEFs from either HOIL-1L^–/–^ or HOIL-1L^*null/null*^ mice (Figure 1F). HOIL-1L has a splice variant, HOIL-N, that contains exons 4 and 5 (Tokunaga et al., 1998) (Figure S1H), and mRNA encoding this region was expressed in cells from the previously established HOIL-1L^–/–^ mice (Figure S1I). Immunoblotting with anti-HOIL-1L serum capable of detecting the N-terminal region, which includes the UBL, revealed that the ~30 kDa HOIL-N fragment was not expressed in HOIL-*1L*^*null/null*^ MEFs, whereas a small amount of HOIL-N was detected in both WT and HOIL-1L^–/–^ MEFs. Moreover, HOIP and SHARPIN were barely detectable in HOIL-1L^*null/null*^ MEFs, in contrast to the previously described HOIL-1L^−/−^ MEFs (Figure 1F). Expression of a mouse HOIL-1L UBL (amino acids [aa] 1–140) in HOIL-1L^*null/null*^ MEFs dramatically increased the amount of SHARPIN and HOIP (Figure 1G) and protected cells against TNF-α-mediated cell death (Figure 1H), implying that the UBL-containing HOIL-N protein may efficiently form a functional trimeric LUBAC complex (Figure S1H). These results clearly demonstrate that the HOIL-1L UBL is essential for HOIP stabilization and that loss of HOIL-1L gene products containing the UBL (HOIL-1L and HOIL-N) profoundly decreased the amount of functional LUBAC in mice and exerted effects comparable to those of loss of HOIP catalytic activity.

### LUBAC Is Stabilized by HOIL-1L and SHARPIN in a Highly Coordinated Manner

**Figure 2.**
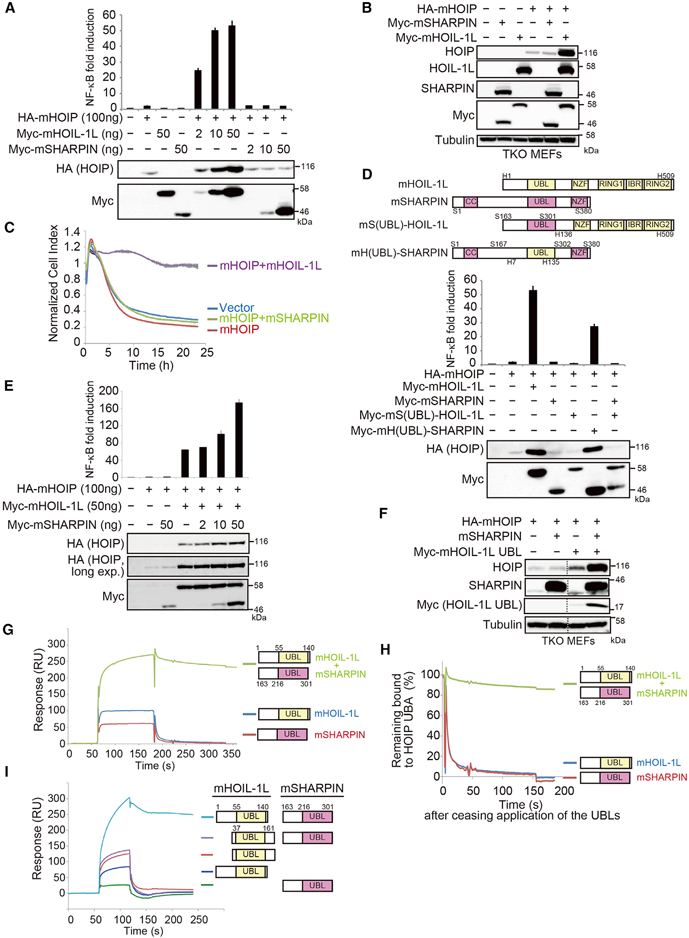
UBLs of HOIL-1L and SHARPIN Bind to HOIP UBA in a Highly Cooperative Manner (A) The indicated expression plasmids and 5× NF-κB luciferase reporters were transfected into HEK293T cells. Cell lysates were probed with indicated antibodies, and NF-κB activity was measured by luciferase assays (means ± SEM, n = 3). (B) Cell lysates of TKO MEFs reconstituted with the indicated proteins were probed as indicated. (C) TKO MEFs expressing the indicated proteins were stimulated with TNF-α (10 ng/ml), and cell viability was measured on a RTCA (mean ± SEM, n = 3). (D) Schematic representation of HOIL-1L, SHARPIN, and their mutants. The indicated expression plasmids and 5× NF-κB luciferase reporters were transfected and analyzed as described in Figure 2A (means ± SEM, n = 3). (E) The indicated plasmids and 5× NF-κB luciferase reporter were transfected into HEK293T cells and analyzed as described in Figure 2A (mean ± SEM, n = 3). (F) Cell lysates of TKO MEFs reconstituted with the indicated proteins were probed as indicated. Vertical dashed lines indicate cropped blots. (G) Glutathione S-transferase (GST)-mHOIP_466–630_ was immobilized on an SPR sensor chip via a GST antibody (Figures 2G and 2I). mHOIL-1L_1–140_ alone (500 μg/ml), mSHARPIN_163–301_ alone (500 mg/ml), or both proteins together (500 mg/ml each) were used as analytes. (H) Plots of data in Figure 2G, with responses normalized against the value at the time application of UBL ceased. (I) Interactions between UBLs containing or lacking the N-terminal region were analyzed as described in Figure 2G. See also Figure S2 and Table S1.

Loss of SHARPIN, another accessory subunit of LUBAC, reduces the level of LUBAC and causes the inflammatory phenotype observed in chronic proliferative dermatitis (cpdm) mice (SHARPIN^cpdm/cpdm^) (Gerlach et al., 2011; Ikeda et al., 2011; Tokunaga et al., 2011). SHARPIN^cpdm/cpdm^ MEFs expressed higher levels of HOIP than did HOIL-1L^*null/null*^MEFs (Figure 1D). Moreover, when co-expressed with mouse HOIP (mHOIP), mSHARPIN failed to increase the amount of mHOIP, efficiently activate NF-κB, or protect cells from TNF-α-mediated cytotoxicity in triple KO (TKO) MEFs lacking all LUBAC subunits, whereas mHOIL-1L could do all three (Figures 2A-2C, S2A, and S2B). Because the homologous UBLs of HOIL-1L and SHARPIN are critical for the interaction with HOIP (Tokunaga et al., 2011), we swapped the UBLs of SHARPIN and HOIL-1L with each other and found that subunits with the HOIL-1L UBL increased the amount of HOIP (Figure 2D). These results indicated that mSHARPIN stabilizes HOIP less effectively than does mHOIL-1L and that the difference between the two subunits can be primarily attributed to their UBLs.

We then evaluated the interactions between the HOIP ubiquitin-associated (UBA) and the UBL of SHARPIN or HOIL-1L using surface plasmon resonance (SPR) analyses. The mSHARPIN fragment containing UBL (aa 163–301; mSHARPIN_163–301_) bound a mHOIP fragment containing the UBA domains (aa 466–630; mHOIP_466–630_) with a calculated dissociation constant (K_D_) of 16.0 ± 2.3 μΜ (Figure S2C; Table S1), a ~10-fold weaker affinity than the interaction between mHOIP_466–630_, and mHOIL-1L containing UBL (aa 1–140; mHOIL-1L_1–140_) (K_D_ = 1.34 ± 0.87 μΜ) (Figure S2D; Table S1). We assumed that the difference in the binding affinity of the HOIP UBA domains for the UBLs underlies the differential roles of the two subunits in LUBAC stabilization.

However, because mSHARPIN increased the amount of mHOIP when co-introduced with mHOIP and mHOIL-1L (Figures 2E and 2F), we suspected that the UBLs play an additional role in LUBAC stabilization beyond their interaction with HOIP. To our surprise, co-application of mHOIL-1L_1–140_ and mSHARPIN_163–301_ led to a much tighter interaction with mHOIP_466–630_ than did either protein alone in the SPR analyses (Figure 2G). Since it is difficult to determine a precise binding constant for interactions among three proteins, we investigated the stabilization by comparing the dissociation of the complexes. The results revealed that, when the two UBL-containing proteins mHOIL-1L_1–140_ and mSHARPIN_163–301_ were applied simultaneously, they remained bound to mHOIP_466–630_ for much longer than either protein alone (Figure 2H). mHOIL-1L_1–140_ and mSHARPIN_163–301_ contain extended N-terminal regions in addition to their UBLs (aa 1–55 and aa 163–206 for HOIL-1L and SHARPIN, respectively). These regions of mHOIL-1L and mSHARPIN share significant sequence similarity (71.8% similarity and 51.3% identity) (Figure S2E). Deletion of the N-terminal region of either mHOIL-1L (aa 37–161; mHOIL-1L_37–161_) or mSHARPIN (aa 198–318; mSHARPIN_198–318_) completely abrogated the cooperative binding to mHOIP_466–630_ in SPR analyses (Figures 2I and S2F). Based on these findings, it seemed plausible that the two accessory subunits are cooperatively involved in formation of trimeric LUBAC via the N-terminal pre-UBLs, in addition to the UBL-mediated interaction with HOIP.

### Structure of the LUBAC Ternary Complex Core Reveals a New Structural Motif Implicated in LUBAC Stabilization

**Figure 3.**
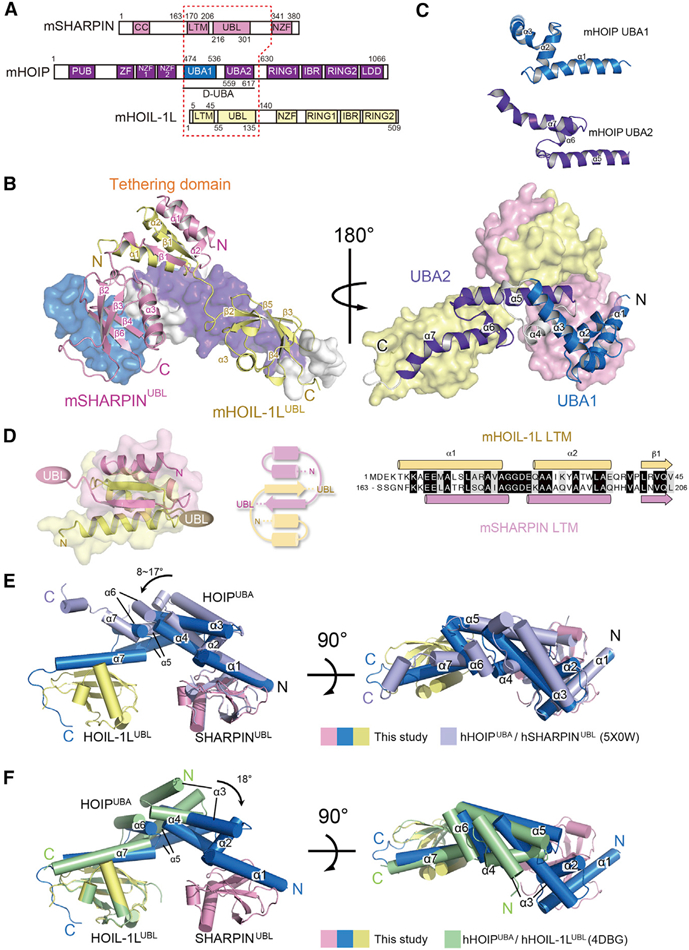
Structure of the LUBAC Ternary Complex Core (A) Schematic representation of the domains of HOIL-1L, HOIP, and SHARPIN. Fragments of mouse subunits used for structural analysis are indicated by a red dotted frame. (B) Overall structure ofthe LUBACternary complex core. The LUBAC core comprises mHOIP D-UBA (aa 474–630) (UBA1, cyan; UBA2, purple; and α4 helix and C-terminal loop, gray), HOIL-1L LTM-UBL(aa1–140) (yellow), and mSHARPIN LTM-UBL (aa 163–341) (pink). Structures are shown from two different viewpoints. (C) Structural comparison between UBA1 and UBA2 ofmHOIP. Helices α–3 and α5–7arefolded into a common UBA module with atypical extended helices α1 and α7. (D) Structure and topological diagram of TD. Sequence alignment of LTMs of mHOIL-1L and mSHARPIN are shown in the right panel. Identical and similar residues are highlighted in black and gray, respectively. (E) Structural comparison between the ternary and hHOIP UBA/hSHARPIN UBL dimeric complexes. The structure of the hHOIP UBA/hSHARPIN UBL complex (PDB: 5X0W) is superposed onto that of the ternary complex core, based on the position of the UBLs ofSHARPIN. The structure ofthe ternary complex core is shown in the same color scheme used in Figure 3A. The representative hHOIP/hSHARPIN binary complex is presented in light purple. (F) Structural comparison between the ternary and hHOIP UBA/hHOIL-1L UBL dimeric complexes. The structure of hHOIP UBA/hHOIL-1L UBL complex (PDB:4DBG) is superimposed ontothat ofthe ternary complex core, based on the UBLs. The structure of the hHOIP/hHOIL-1L complex is shown in green. See also Figures S3 and S4 and Table S2.

Previous structural studies of the heterodimeric core regions of LUBAC have identified the individual sites in human HOIP (hHOIP) involved in the interactions with hHOIL-1L UBL and hSHARPIN UBL (Liu et al., 2017; Yagi et al., 2012). The UBLs of hHOIL-1L and hSHARPIN simultaneously associate with the different UBA-like modules in the hHOIP UBA regions, but in completely different binding modes (Liu et al., 2017). However, detailed structural information of the ternary complex core of LUBAC is essential for understanding how the three subunits are assembled and stabilized in the fully active form and especially for determining the roles of the N-terminal pre-UBLs of HOIL-1L and SHARPIN in stable LUBAC formation. Hence, we determined a crystal structure of mHOIP_474–630_ in ternary complex with mHOIL-1L_1–140_ and mSHARPIN_163–341_ at 2.4 Å resolution (Figures 3A and 3B; Table S2). As estimated via analytical size-exclusion chromatography (Figure S3A–S3C), the core subunits assemble into a complex with 1:1:1 stoichiometry. In the ternary complex, mHOIP_474–630_ adopts an elongated configuration comprising seven *a* helices and two 3_10_ helices and contains two UBA-like modules, the N-module (aa 474–536; UBA1) and the C-module (aa 559–617; UBA2), tandemly linked through a middle helix α4 (Figures 3B and 3C). Therefore, mHOIP_474–630_ is hereafter designated as a double-UBA (D-UBA) domain. UBA1 and UBA2 exhibit completely distinct UBL-binding modes (Figure S3D). The N-terminal μ1 helix in UBA1 and the C-terminal μ7 helix in UBA2 provide the major binding sites for the UBLs of SHARPIN and HOIL-1L, respectively; consequently, in the ternary complex, the two UBLs are independently arranged at opposite ends of the elongated mHOIP D-UBA.

Furthermore, the crystal structure of the LUBAC ternary complex core has a new structural motif common to the pre-UBLs of mHOIL-1L and mSHARPIN (residues 5–45 of mHOIL-1L and 170–206 of mSHARPIN), which possess a unique helix-helix-strand structure that mediates stable heterodimerization. Accordingly, we termed these regions ‘‘LUBAC-tethering motifs (LTMs).’’ Intriguingly, the pair of LTMs folds into a single globular domain, referred to as the ‘‘tethering domain (TD)’’ (Figures 3B and 3D). Owing to their high sequence similarity, the two LTMs in the TD are related by a pseudo-2-fold axis. The HOIL-1L LTM is linked to its UBL via a long flexible loop, whereas the SHARPIN LTM is connected to its UBL via a 3_10_ helix. The TD and HOIP D-UBA undergo no significant interaction, except for a small contact site that could be attributed to crystal packing effects. Thus, the TD is structurally independent of the other domains of LUBAC. The formation of the TD supports the idea that both LTMs are required for stable assembly of the LUBAC ternary core, as indicated by our SPR analyses (Figures 2I and S2F).

Next, we compared our trimeric LUBAC core structure with the previously reported structures of the hHOIP/hHOIL-1L and hHOIP/hSHARPIN dimeric cores. The manner of the interaction between the mHOIP UBA1 and mSHARPIN UBL in the ternary complex is very similar to that observed in the hHOIP UBA/hSHARPIN UBL dimeric complex (Figures S3D and S3E) (Liu et al., 2017). The non-canonical UBA-UBL interaction mode between the mHOIP UBA2 and mHOIL-1L UBL is also identical to that observed in the hHOIP UBA/hHOIL-1L UBL dimer (Figures S3D and S3E) (Yagi et al., 2012). Thus, the pair of UBA-UBL interactions in the ternary complex is not influenced by LTM-mediated heterodimerization of mSHARPIN and mHOIL-1L. On the other hand, mHOIP D-UBA in the ternary complex adopts a conformation different from that of the hHOIP D-UBA bound to either hSHARPIN UBL or hHOIL-1L UBL. Specifically, superimposition of mHOIP UBA1 in the ternary complex onto hHOIP UBA1 in the hHOIP UBA/hSHARPIN UBL dimer revealed a different spatial arrangement of UBA2 (Figure 3E). Similarly, the arrangement of UBA1 differs between the ternary complex and the hHOIP UBA/hHOIL-1L UBL dimer when their UBA2 were superimposed onto each other (Figure 3F). No significant interaction between UBA1 and UBA2 is caused by simultaneous binding of UBLs of mHOIL-1L and mSHARPIN. Taken together, these findings suggest that tethering the UBLs of SHARPIN and HOIL-1L via their LTMs induces a more compact and rigid conformation of HOIP D-UBA than could be achieved by either UBL alone.

### Functional Differences between Human and Mouse HOIP UBA in LUBAC Stabilization

The molecular interface between the mHOIP UBA1 and mSHARPIN UBL in the ternary complex looked almost identical to that of hHOIP D-UBA and hSHARPIN, as described above (Figures S3D and S3E). The conserved hydrophobic patch of the SHARPIN UBL is mainly recognized by the extended helix α1 in HOIP UBA1, and recognition involves coordinated hydrophobic and electrostatic interactions (Figure S3F). However, we suspected functional differences between the mouse and human HOIP UBA/SHARPIN UBL interfaces, because mSHARPIN failed to increase the level of mHOIP (Figure 2), whereas hSHARPIN could do so (Gerlach et al., 2011; Ikeda et al., 2011; Tokunaga et al., 2011). We confirmed that the amount of hHOIP, but not mHOIP, was increased by SHARPIN regardless of its species origin (Figure S4A). We attributed this effect to the difference in the D-UBA, as evidenced by the observation that mSHARPIN could increase the level of a mHOIP mutant containing the human D-UBA (hD-UBA) (Figures S4B and S4C).

A detailed structural comparison suggested that hHOIP R496 would be more advantageous for a stable UBA-UBL interface than would be the corresponding residue of mHOIP, Q490 (Figure S4B); the side chain of hHOIP R496 forms electrostatic interactions with a side-chain carbonyl group of E226 and a main-chain carboxyl group of D227 and strengthens the association with the acidic region in the vicinity of the conserved hydrophobic patch in SHARPIN UBL (Figure S4D). Importantly, luciferase analyses confirmed that the functional difference between hHOIP/hSHARPIN and mHOIP/mSHARPIN in LUBAC stabilization can be mainly attributed to this amino acid difference (Figure S4E). This residue is Q in rodents (mouse and rat), but R in cattle, chimpanzees, and humans (Figure S4F). Thus, a single amino acid substitution that arose over the course of evolution potentiates the association between HOIP UBA and SHARPIN UBL via electrostatic interactions, thereby substantially stabilizing the SHARPIN-HOIP complex.

### Co-folding of the LTMs of HOIL-1L and SHARPIN Plays a Crucial Role in Trimeric LUBAC Stabilization and Function

**Figure 4.**
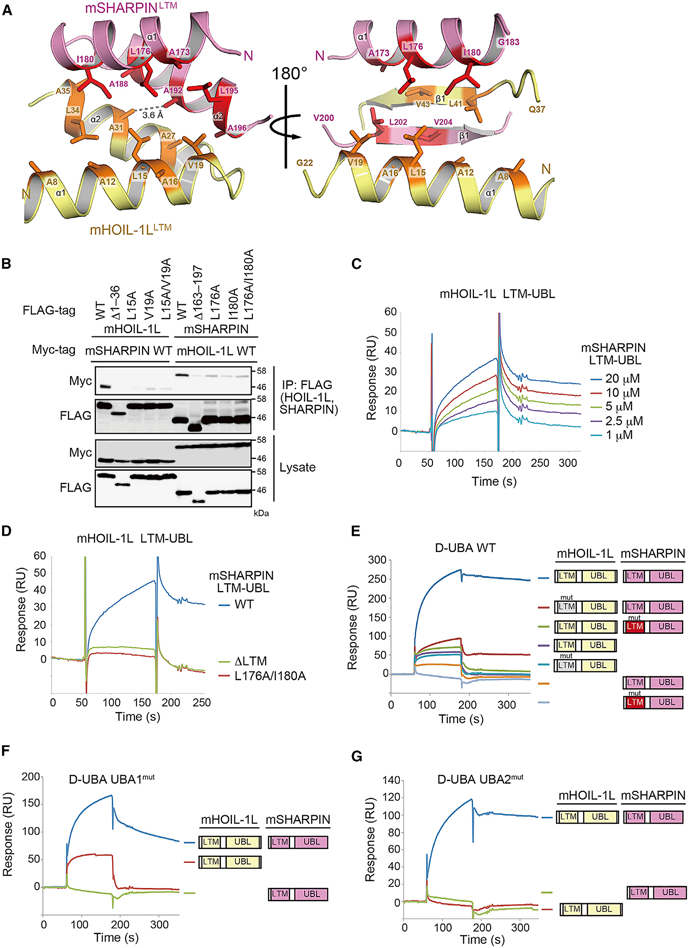
Novel HOIL-1L-SHARPIN Interactions Mediated by LTMs (A) Heterodimeric interface ofTD formed by LTMsofmHOIL-ILand mSHARPIN. The residuesforming the hydrophobiccore of TD areshown asstick models and highlighted in red (mSHARPIN) or orange (mHOIL-1L). β1 strands and α2 helices of both molecules are eliminated from the ribbon models in the left and right panels, respectively. (B) The indicated expression plasmidsweretransfected into HEK293T HOIP KO cells. Cell lysates and anti-FLAG immunoprecipitateswere probed as indicated. (C) mHOIL-1L_1–189_-strepwas immobilized on a SPR sensorchip with anti-strep antibody. Binding between mHOIL-1L_1–189_ and MBP-mSHARPIN UBL_163–301_ was evaluated. (D) Interactions between mHOIL-1L_1–189_ and MBP-mSHARPIN mutants were analyzed as described in Figure 4C. mSHARPIN_163–301_ WT, L176A/I180A, and mSHARPIN_198–318_ ΔLTM (40 mM) were used as analytes. (E-G) GST-mHOIP D-UBA (aa 466–630) WT (E), UBA1^mut^ (F), and UBA2^mut^ (G) were immobilized on a SPR sensor chip with anti-GST antibody. Binding to UBLs of WT or LTM^mut^ was analyzed like in Figure 2G. See also Figure S5 and Table S1.

The major heterodimeric interface in the TD is formed by the hydrophobic faces of the α1 and α2 helices from both LTMs and an intermolecular antiparallel β sheet consisting of the β1 strands (aa 41–45 of mHOIL-1L and 202–206 of mSHARPIN) (Figure 4A). In the TD, A31 of mHOIL-1L and A192 of mSHARPIN in the α2 helices make a direct van der Waals contact with each other (Figure 4A). Consistent with this observation, the A31F mutation and the combination of mHOIL-1L A31D and mSHARPIN A192D also diminished the LTM interaction when the mutant proteins were expressed in HEK293T HOIP KO cells (Figure S5A–S5C). Moreover, alanine substitution of the hydrophobic residues in the α1 helices, L176 and I180 of mSHARPIN or L15 and V19 of mHOIL-1L, significantly diminished binding compared with the WT proteins (Figure 4B). Application of SHARPIN_163–301_ (LTM-UBL) to the sensor tip, while fixing HOIL-1L_1–189_ (LTM-UBL), revealed that the LTM-UBLs of HOIL-1L and SHARPIN formed a complex, but the introduction of the L176A/I180A mutations to SHARPIN abolished the interaction (Figures 4C and 4D). Thus, HOIL-1L and SHARPIN can bind each other directly; the K_D_ of the HOIL-1L-SHARPIN interaction was calculated as 2.04 ± 0.29 μM (Figure 4C; Table S1).

It is of note that the dissociation kinetics of LTM-mediated interactions were slower than those of the interactions between the D-UBA of HOIP and the UBL of HOIL-1L or SHARPIN (Figures 2G, 2H, 4C, S2C, and S2D). Hence, we performed SPR analyses to assess the contributions of the three interactions between LUBAC subunits to the formation and stabilization of LUBAC. Mutations of amino acids affecting LTM-mediated dimerization in either HOIL-1L (L15A/V19A: HOIL-1L LTM^mut^) or SHARPIN (L176A/I180A: SHARPIN LTM^mut^) almost completely abolished the formation of the stable trimeric LUBAC core (Figure 4E). By contrast, introduction of mutations in UBA1 (UBA1^mut^ [R474A/L483A/V486A]) or UBA2 (UBA2^mut^ [Q607A/L611A/F614A]) of HOIP D-UBA, which impaired the SHARPIN-HOIP or HOIL-1L-HOIP interaction, respectively, only marginally attenuated dissociation of trimeric LUBAC core (compare Figures 4F and 4G with 4E). These results strongly indicate that the LTM-mediated HOIL-1L/SHARPIN interaction is critical for stable trimeric LUBAC formation.

**Figure 5.**
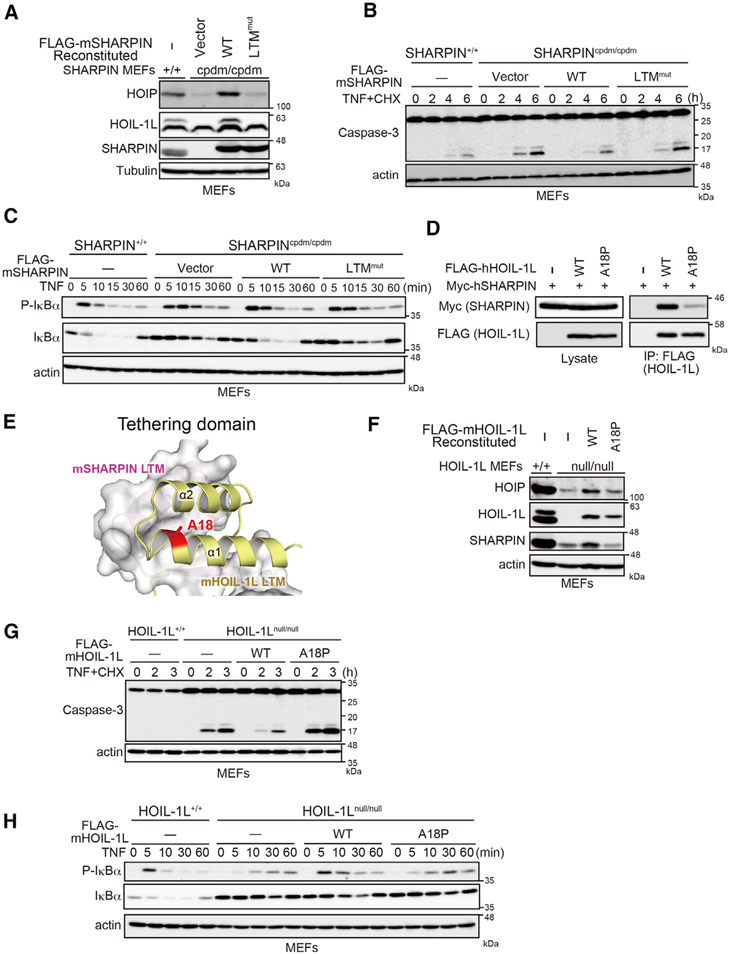
Crucial Role of LTM-Mediated HOIL-1L/SHARPIN Interaction in Trimeric LUBAC Formation and Stabilization (A) Immunoblot analysis of lysates from WT or cpdm MEFs reconstituted with the indicated proteins. (B and C) cpdm MEFs stably reconstituted with the indicated proteins were stimulated with TNF-α (10 ng/ml) plus CHX (20 mg/ml) (B) or TNF-α (10ng/ml)(C) fortheindicated periods, followed by immunoblotting. (D) The indicated expression plasmids were transfected into HEK293T HOIP KO cells. Cell lysates and anti-FLAG immunoprecipitates were immunoblotted as indicated. (E) LTMs of HOIL-1L and SHARPIN are shown as a ribbon model and on the molecular surface, respectively. Ala18 of HOIL-1L (red) is located at the surface of the TD. (F) Cell lysates of HOIL-1L-null MEFs stably expressing the indicated proteins were probed as indicated. (G and H) HOIL-IL-null MEFs stably expressing the indicated proteins were stimulated with TNF-α (10 ng/ml) plus CHX (20 mg/ml) (G) or TNF-α (10 ng/ml) (H) for the indicated periods, and cell lysates were analyzed by immunoblotting. See also Figure S5.

Next, we evaluated the roles of the LTM-mediated HOIL-1L/SHARPIN interaction in stable LUBAC formation and physiological functions. Although mSHARPIN WT increased the level of mHOIP, mSHARPIN L176A/I180A (LTM^mut^) failed to do so when retrovirally introduced into SHARPIN-null cpdm MEFs (Figure 5A). In accordance with the level of mHOIP, cpdm MEFs expressing mSHARPIN LTM^mut^, but not WT, could not prevent caspase-mediated cell death following stimulation with TNF-α plus cycloheximide (CHX) or effectively induce degradation of IκBa, a hallmark of NF-κB activation, in response to TNF-α (Figures 5B and 5C). Furthermore, biallelic mutation of HOIL-1L A18P, located within the HOIL-1L LTM, has been reported in patients with polyglucosan body myopathy (Nilsson et al., 2013). The A18P mutation in mouse or human HOIL-1L dramatically attenuated the HOIL-1L/SHARPIN interaction (Figures 5D and S5D). Our structural analyses indicated that substitution of Ala18 with Pro might change the structure near the helix α1-loop region of HOIL-1L LTM (Barlow and Thornton, 1988), potentially leading to local perturbation of helix bundle formation, and thereby weakening the hydrophobic core interface of the TD (Figure 5E). Indeed, HOIL-1L A18P failed to effectively increase the amount of HOIP and SHARPIN when introduced into HOIL-1L^*null/null*^ MEFs (Figure 5F). Following stimulation with TNF-α plus CHX, active caspase-3 was detected at a much higher level in HOIL-1L^*null/null*^ MEFs expressing the HOIL-1L A18P mutant than in those expressing HOIL-1L WT (Figure 5G). Moreover, the A18P mutant upregulated NF-κB activation by TNF-α much less efficiently than HOIL-1L WT (Figure 5H). These observations indicate that the LTM-mediated HOIL-1L/SHARPIN interaction plays a central role in stabilization and function of trimeric LUBAC.

### Therapeutic Potential of Targeting the HOIL-1L/SHARPIN Interaction

**Figure 6.**
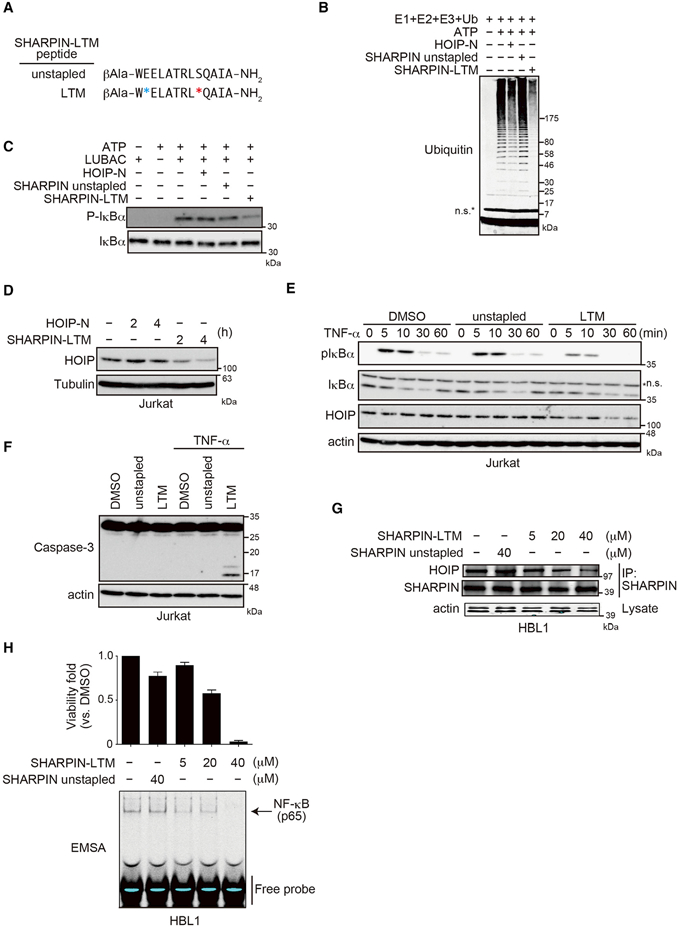
Targeting the HOIL-1L/SHARPIN Interaction Using an α-Helical-Stapled Peptide (A) Sequences of α-helical stapled SHARPIN peptides. Asterisks show the locations of the hydrocarbon cross-linker. (B) Trimeric LUBAC (0.2 μM) was incubated with stapled peptides (80 μM) on ice for 3 hr. A mixture of E1, E2, and ubiquitin was added, and the sample was incubated at 37°C for 30 min, followed by immunoblotting with anti-ubiquitin. (C) S100 lysates of Jurkat HOIP KO cells (10 mg) and trimeric LUBAC (0.1 mM) were incubated with stapled peptides (80 mM) on ice for 3 hr, followed by incubation with a mixture of E1, E2, and ubiquitin at 37°C for 30 min, and were probed by immunoblotting. (D) Jurkat cells were treated with the indicated peptides (20 mM) for the indicated periods. Cell lysates were probed by immunoblotting. (Eand F) Jurkat cells were treated with the indicated peptides (20 mM) for 2 hr, followed by stimulation with TNF-α (10 ng/ml) for the indicated periods (E) or 4 hr (F). Cell lysates were probed by immunoblotting. (G) Cell lysates and anti-SHARPIN immunoprecipitates from HBL1 cells treated with the indicated peptides for 24 hr. were probed by immunoblotting. (H) HBL1 cells were treated with the indicated peptides for 24 hr. Cell viability was measured using the CellTiter-Glo Luminescent Cell Viability assay, (mean ± SEM, n = 3) and NF-κB activity was measured by EMSA. See also Figure S6.

We previously reported that augmented LUBAC ligase activity is involved in the pathogenesis of the activated B cell-like type of diffuse large B cell lymphoma (ABC-DLBCL) and that inhibition of the HOIP/HOIL-1L interaction by a hydrocarbon-stapled a-helical peptide (HOIP-N) can suppress the proliferation of ABC-DLBCL cells (Yang et al., 2014). Given the crucial involvement of the LTM-mediated HOIL-1L/SHARPIN interaction in LUBAC stability and function, we generated an a-helical stapled peptide mimicking the LTM of SHARPIN (SHARPIN-LTM) to inhibit the HOIL-1L/SHARPIN interaction and destabilize the pre-existing LUBAC complex (Figure 6A). The SHARPIN-LTM peptide inhibited LUBAC ligase activity and IKK activation more effectively than HOIP-N, as demonstrated by *in vitro* assays (Figures 6B and 6C). More importantly, the SHARPINLTM peptide destabilized HOIP more efficiently than HOIP-N in Jurkat cells (Figure 6D). SHARPIN-LTM also inhibited LUBAC functions, namely, suppression of NF-κB activation (as evaluated by phosphorylation and degradation of IκBa) and promotion of caspase-mediated cell death induced by TNF-α (Figures 6E and 6F).

We then examined the therapeutic potential of inhibition of the HOIL-1L/SHARPIN interaction for treating ABC-DLBCL, using HBL1 cells derived from lymphoma patients. The SHARPINLTM peptide reduced the amount of the LUBAC complex (Figure 6G), thereby profoundly impairing TNF-α-induced IκBa degradation, NF-κB activation, and secretion of the NF-κB target interleukin-8 (IL-8) (Figures 6H and S6A–S6C). Finally, we examined the effect of SHARPIN-LTM on the fate of HBL1 cells, in which proliferation is dependent on LUBAC (Yang et al., 2014). Inhibition of the HOIL-1L/SHARPIN interaction caused HBL1 cells to undergo cell death (Figure 6H). These results clearly demonstrate that inhibition of the HOIL-1L/SHARPIN interaction can kill ABC-DLBCL cells, suggesting a novel therapeutic approach against this type of lymphoma.

## DISCUSSION

**Figure 7.**
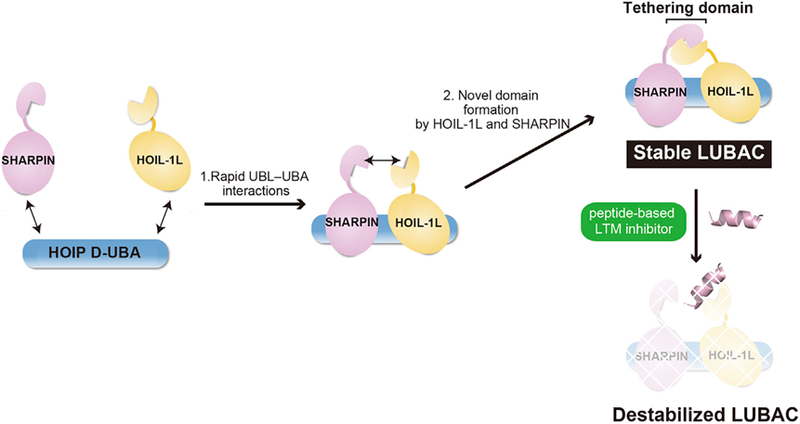
Mechanisms Underlying Trimeric LUBAC Stabilization HOIL-1L and SHARPIN gather on the HOIP UBA via rapid interactions between HOIP UBA and UB. During this process, the LTMs of both proteins heterodimerize to form the TD. Peptidebased LTM inhibitor destabilizes LUBAC by blocking TD formation.

LUBAC forms a stable complex comprising three subunits. In this study, we determined the crystal structure of the trimeric LUBAC core at 2.4 Å resolution. This structure convincingly demonstrates that the three subunits interact with each other, and that the three inter-subunit interactions in the trimeric core make critical contributions to overall stabilization of LUBAC. Among the three interactions, two (between HOIP and HOIL-1L or SHARPIN) are mediated by atypical UBA-UBL interactions (Figures 3B and S3D). In addition, our ternary complex structure shows that newly identified LTM motifs in two accessary subunits are co-folded into a single domain that forms the essential molecular interface for the LUBAC assembly (Figures 3D and 4A). The mSHARPIN/mHOIL-1L interface possesses a larger buried surface area (1,419 Å) than UBA-UBL interfaces (1,126 A for mHOIP/mSHARPIN; 862.6 Å for mHOIP/mHOIL-1L) and should therefore be more stable (Chen et al., 2013). Consistently, SPR analyses clearly indicated that the LTM-mediated SHARPIN/HOIL-1L interaction was resistant to dissociation (Figure 4C), whereas the two UBA-UBL interactions associated and dissociated more rapidly (Figures 2G, 2H, S2C, and S2D). We previously observed that HOIP facilitates the interaction between SHARPIN and HOIL-1L when transiently expressed in HEK293T cells (Tokunaga et al., 2011). Thus, the two UBL-UBA interactions and the LTM-mediated interaction play different roles in the formation and stabilization of LUBAC. First, the three subunits gather via interactions between HOIP D-UBA and UBLs of HOIL-1L and SHARPIN. Subsequently, TD is stably formed by heterodimerization of LTMs from HOIL-1L and SHARPIN (Figure 7). Once TD forms on HOIP D-UBA, the two UBL domains become resistant to dissociation from HOIP. We presume that this enhancement of binding is due to an avidity effect: even if one of the two UBL-UBA interactions is lost, the UBL itself does not dissociate from HOIP D-UBA because another UBL-UBA interaction and TD prevent the UBL from diffusing away, and binding will eventually be restored. Furthermore, HOIL-1L/SHARPIN dimerization appears to promote conformational stability and integrity of the LUBAC core by securing simultaneous binding of two UBLs to HOIP. The D-UBAs in the binary hHOIP/hSHARPIN and hHOIP/hHOIL-1L complexes adopt different conformations (Liu et al., 2017; Yagi et al., 2012), implying that this domain has some structural flexibility. The C-terminal extended α7-helix formation in HOIP D-UBA is induced upon binding of the HOIL-1L UBL in the trimeric and hHOIP/hHOIL-1L dimeric core structures (Figures 3E and 3F). Furthermore, the HOIP UBA2 in the ternary complex is rotated by 8°−17° in comparison with that in the hHOIP/hSHARPIN dimer (Figure 3E). Simultaneous binding of both UBLs of SHARPIN and HOIL-1L is likely to facilitate the compact configuration of HOIP D-UBA, which is presumably required for proper catalytic activity of LUBAC. Heterodimerization of HOIL-1L and SHARPIN via their LTMs may promote the co-existence of two UBLs on HOIP D-UBA, and thereby contribute to maintaining the ternary LUBAC core in an appropriate conformation. Indeed, the dissociation rate constant (k_diss_) of the trimetric LUBAC core (k_diss_ = 0.000111 s^−1^) was comparable to that of the HOIL-1L/SHARPIN complex (k_diss_ = 0.00361 s^−1^) (Figure 4C). Thus, formation of a single domainlike structure from two peptides from different proteins is an effective strategy for forming a stable complex. Moreover, our observation that biallelic mutation of A18P in the LTM of HOIL-1L causes polyglucosan body myopathy by drastically reducing the amount of LUBAC highlights the pathophysiological significance of the LTM-mediated HOIL-1L-SHARPIN heterodimerization (Figures 5D–5H). Other examples of the formation of TD-like domains in heteromeric protein-protein interactions might be reported in the future.

Loss of either HOIL-1L or SHARPIN, abolishes two out of three interactions (i.e., one UBL-UBA and one LTM mediated) and profoundly destabilizes LUBAC. However, the outcome of such a loss differs depending on the subunit: loss of HOIL-1L causes embryonic lethality, whereas loss of SHARPIN causes autoinflammation and immunodeficiency in mice. This discrepancy might be attributed to differences in the ability to stabilize catalytic HOIP (Figure 2). The dissociation constant (K_D_) of the mHOIL-1L-mHOIP is 10-fold lower than that of the mSHARPIN-mHOIP interaction (Figures S2C and S2D; Table S1). Consistent with this, mHOIL-1L could increase the amount of mHOIP, but mSHARPIN failed to do so. However, the amount of the catalytic HOIP is not the sole determinant of phenotype. HOIL-1L^−/−^ mice that express the HOIL-N alternative splicing product exhibit only glycogen-like deposits in muscles (MacDuff et al., 2015; Shimizu et al., 2016), whereas SHARPIN-null cpdm mice have more severe symptoms than HOIL-1L^−/−^ mice despite having similar amounts of HOIP. Thus, the composition of the LUBAC complex should also be taken into account, because LUBAC containing SHARPIN inhibits programmed cell death induced by TNF-α more effectively than LUBAC lacking SHARPIN (Shimizu et al., 2016).

In human, patients with N-terminal mutations in HOIL-1L who apparently lack both HOIL-N and HOIL-1L are viable but suffer from immunodeficiency and autoinflammation (Boisson et al., 2012), whereas HOIL-1L^*null/null*^ mice that lack both products exhibit embryonic lethality (Figure 1). The phenotypic differences between mouse and human may be attributable to the differences between the D-UBAs of mHOIP and hHOIP (Figures S4B and S4C). The single amino acid substitution of Q490 in mHOIP to R496 in hHOIP potentiates the association between HOIP UBA1 and SHARPIN UBL via an electrostatic interaction, thereby substantially stabilizing the hSHARPIN/hHOIP complex (Gerlach et al., 2011; Ikeda et al., 2011; Tokunaga et al., 2011). Therefore, trace amounts of LUBAC comprise SHARPIN and HOIP and might be present in patients with immunodeficiency or autoinflammation, allowing these patients to be viable.

As shown in Figure 6, a reduction in the LUBAC level is a promising therapeutic strategy for treating ABC-DLBCL (Yang et al., 2014). Accordingly, agents capable of inducing destabilization of trimeric LUBAC could be used against ABC-DLBCL or other LUBAC-dependent cancers. Of the three interactions between LUBAC subunits, the newly identified LTM-mediated HOIL-1L/SHARPIN dimerization appears to play a predominant role in stabilizing LUBAC, as demonstrated by our SPR analyses (Figures 2G-2I and 4E-4G). The amino acid sequences of LTMs are highly conserved among HOIL-1L and SHARPIN (Figures S5E and S5F), implying that stabilization of LUBAC by LTM-mediated interactions between HOIL-1L and SHARPIN is a general mechanism. Thus, the SHARPIN/HOIL-1L interaction seems to be the most promising therapeutic target among the three interactions (Figure 7). Reduction of HOIP by disruption of trimeric LUBAC appears to be mediated via the ubiquitin-proteolytic pathway, because an inhibitor of ubiquitin E1 (MLN-7243), but not lysosomal proteases inhibitors (E64d/pep), could suppress decrease of HOIP in MEFs lacking the SHARPIN subunit of LUBAC (Figure S6D). Loss of LUBAC ligase activity leads to embryonic lethality (Shimizu et al., 2016); however, LUBAC ligase activity is not completely abolished by inhibition of the SHARPIN/HOIL-1L interaction because it does not affect the other interactions between the LUBAC components, and LUBAC containing HOIL-1L/HOIP or SHARPIN/HOIP can exist in humans. Therefore, agents that target the SHARPIN/HOIL-1L interaction could have fewer side effects than do other anticancer drugs. In addition to the crucial roles of LUBAC in the oncogenesis of ABC-DLBCL (Yang et al., 2014), LUBAC activity is also involved in resistance to immune checkpoint blockade therapy and cisplatin (MacKay et al., 2014; Manguso et al., 2017). Thus, the crucial role of LTM-mediated heterodimerization of the two accessory subunits in stable formation of trimeric LUBAC suggests a therapeutic strategy for the treatment of these malignant tumors.

## EXPERIMENTAL PROCEDURES

Further details and an outline of resources used in this work can be found in the Supplemental Experimental Procedures and in Table S3.

### Mice

Fertilized oocytes were microinjected with pX330 containing a guide RNA sequence against HOIL-1L (Figure S1A). Progeny were genotyped using the following primers: typing_Fwd, 5′-TTGCCAACAGGCCAATTTGATG-3′ and typing_Rev, 5′-TGCGGTGATGCACAATATCCTG-3′. For timed mating of mice, a single male was mated with one or two females. The day that a vaginal plug was detected was considered as E0.5. All mice were maintained under specific pathogen-free conditions in the animal facilities of Kyoto University. All animal protocols were approved by Kyoto University.

### Crystallization, Data Collection, and Structural Determination

Crystallization was performed by the sitting-drop vapor diffusion method at 20°C. Crystals of the LUBAC ternary complex core were grown from drops consisting of 200 nL of protein solution (7.1 mg/ml) and 100 nL of reservoir solution containing 1.8 M magnesium sulfate and 0.1 M MES (pH 6.5). For X-ray diffraction measurements, the crystals were cryoprotected in reservoir solution supplemented with 25% glycerol.

X-ray diffraction datasets were collected at 95 K on beamlines BL-17A at the Photon Factory (Tsukuba, Japan). Diffraction data were processed using HKL2000 (Otwinowski and Minor, 1997). Data collection statistics are summarized in Table S2.

The structure of the LUBAC ternary complex core was determined by the molecular replacement method using Molrep (Vagin and Teplyakov, 1997) in the CCP4 suite (Winn et al., 2011), using the coordinates of the complex of human HOIP D-UBA and HOIL-1L UBL (PDB ID: 4DBG) as a search model. The initial model was built automatically using Buccaneer (Cowtan, 2006), and subsequent model building was performed manually using COOT (Emsley et al., 2010). Structural refinement was conducted using Refmac (Murshudov et al., 1999). The statistics of structural refinement and the quality of the final model are summarized in Table S2. Secondary structure assignment of each LUBAC subunit was performed using DSSP (Touw et al., 2015). All figures depicting the crystal structure were produced using PyMol (https://pymol.org/2/).

### Qualification and Statistical Analysis

Dissociation constant values of SPR measurements are presented as ± SD. Other data are presented as means ± SEM. In Figure S1F, statistical analysis was performed using GraphPad Prism (v.5.). Comparisons were performed with a two-tailed Student’s t test, and p values are represented in figures as ** p < 0.01.

## Supplementary Material

Supplement

## Highlights

- The crystal structure of trimeric LUBAC core solved at 2.4 Å resolution
- Formation of a single domain by HOIL-1L and SHARPIN stabilizes LUBAC
- Targeting the HOIL-1L/SHARPIN interaction efficiently disrupts LUBAC
- Loss of the HOIL-1L UBL in mice leads to embryonic lethality
