# Supplementary material for: Cooperative Domain Formation by Homologous Motifs in HOIL-1L and SHARPIN Plays A Crucial Role in LUBAC Stabilization
Source: Cell Rep. Author manuscript; Available in PMC 2019 Apr 24. (PMC6044281; doi:10.1016/j.celrep.2018.03.112)
Supplement: Supplement [file NIHMS975286-supplement-Supplement.pdf]

Cell Reports, Volume *23*

Supplemental Information

Cooperative Domain Formation by Homologous Motifs in HOIL-1L and SHARPIN Plays A Crucial Role in LUBAC Stabilization

Hiroaki Fujita, Akira Tokunaga, Satoshi Shimizu, Amanda L. Whiting, Francisco Aguilar-Alonso, Kenji Takagi, Erik Walinda, Yoshiteru Sasaki, Taketo Shimokawa, Tsunehiro Mizushima, Izuru Ohki, Mariko Ariyoshi, Hidehito Tochio, Federico Bernal, Masahiro Shirakawa, and Kazuhiro Iwai

A

HOIL-1L 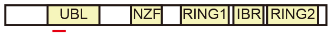

guideRNA #1 : AGTACGCCCGGATATGACAG

guideRNA #2 : CATTTGGCTCACAGTACGCC

B

WT sequence: acagtacgcccggatatgacagtggcctcccttaag

allele #1 (null): acagtacgcccggatatga==gtggcctcccttaag

allele #2 (null#2): acagtaccgcccggatatgacagtggcctcccttaag

C

HOIL-1L$^{+/null\#2}$ × HOIL-1L$^{+/null\#2}$	HOIL-1L$^{+/+}$	HOIL-1L$^{+/null\#2}$	HOIL-1L$^{null\#2/null\#2}$	total
No. of mice	28	41	0	69

D

HOIL-1L$^{+/null\#2}$ × HOIL-1L$^{+/null\#2}$

	HOIL-1L$^{+/+}$	HOIL-1L$^{+/null\#2}$	HOIL-1L$^{null\#2/null\#2}$	total
E9.5	4	9	2	15
E10.5	5	7	2	14
E11.5	7	16	0	23
E12.5	4	9	0	13

E

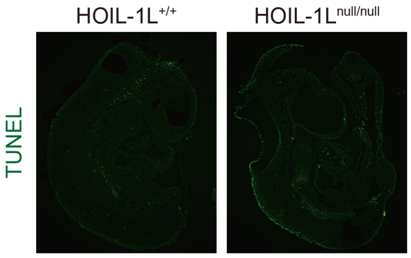

F

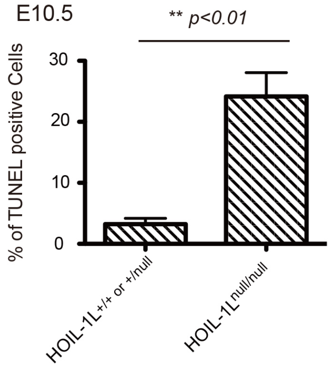

G

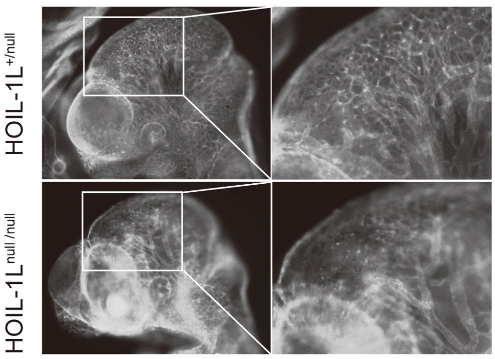

H

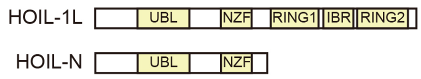

I

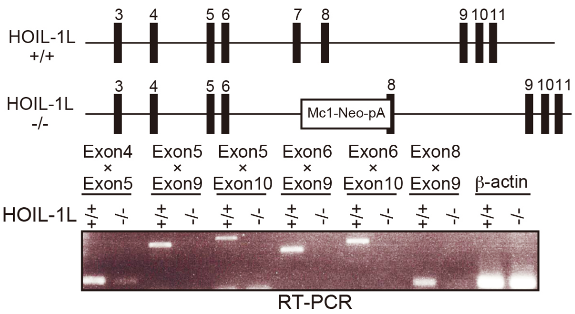

Figure S1. Generation of HOIL-1L–null mice and their phenotype (Related to Figure 1)

(A) Guide RNA sequences against mHOIL-1L used for generation of knockout mice.

(B) Mutated allele #1 was obtained using guide RNA#1, and mutated allele #2 was obtained using guide RNA#2.

(C) Number of offspring of each genotype obtained by crossing HOIL-1L$^{+/null\#2}$ mice.

(D) Numbers of embryos obtained at each embryonic stage (E9.5, 10.5, 11.5, and 12.5) by crossing HOIL-1L$^{+/null\#2}$ mice.

(E and F) Representative images of TUNEL staining of paraffin-sectioned embryos of the indicated genotypes at E10.5 (E) and quantification of TUNEL-positive cells in embryos of the indicated genotypes (n = 3) (F). Data are expressed as means ± s.e.m. (Significance: **, $p < 0.01$).

(G) Representative images of whole-mount staining of embryos of indicated genotypes with anti-CD31 antibody at E10.5.

(H) Schematic representation of domains of HOIL-1L and HOIL-N.

(I) RT-PCR analysis of expression levels of the N-terminal and C-terminal parts of HOIL-1L in MEF cells derived from HOIL-1L$^{-/-}$ or WT ($^{+/+}$) mice.

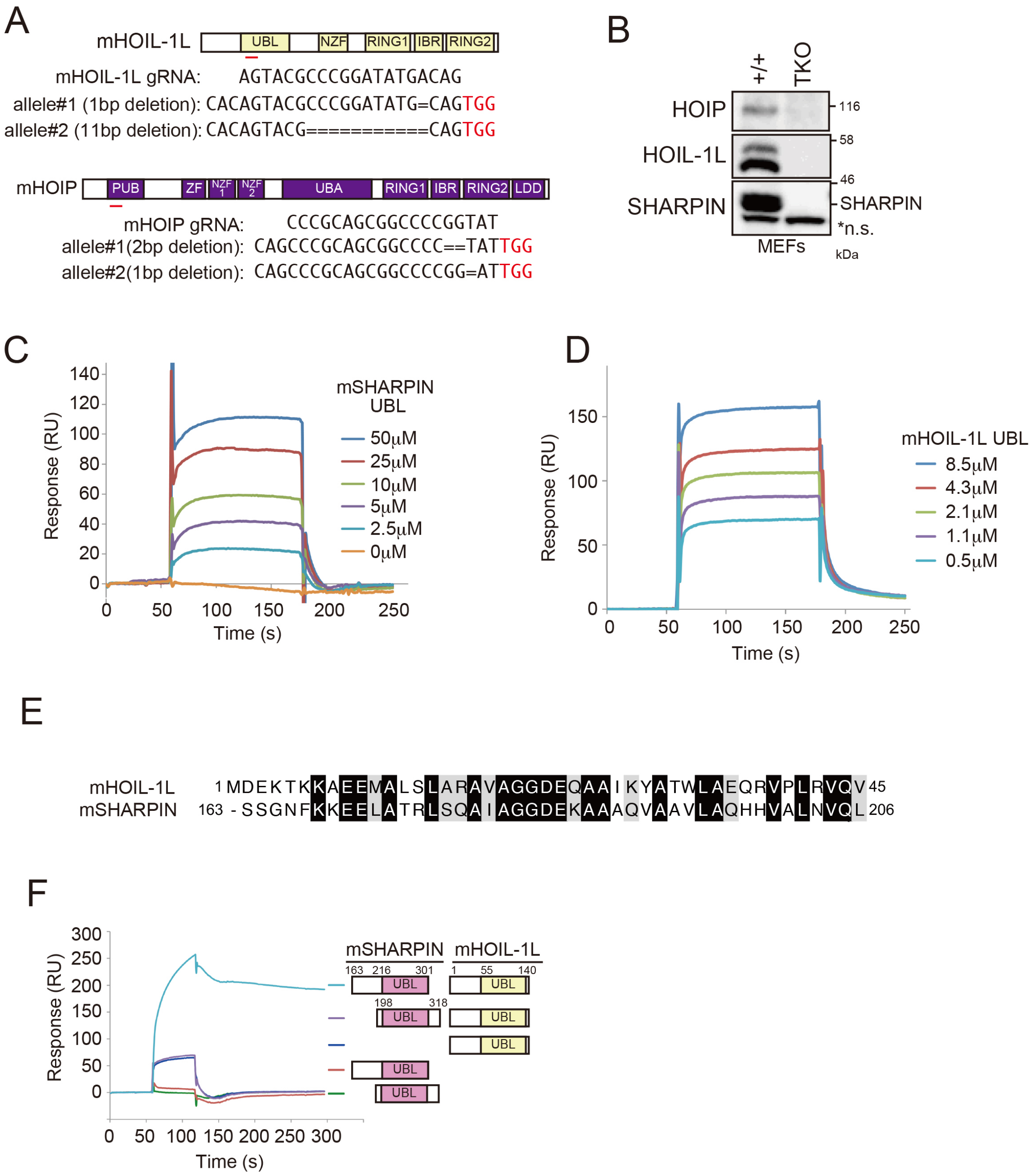

Figure S2. Role of HOIL-1L and SHARPIN in LUBAC stabilization (Related to Figure 2)

(A) Guide RNA sequences against mHOIP or mHOIL-1L. TKO MEFs carry one allele with a 1 bp deletion and one allele with an 11 bp deletion in the HOIL-1L locus, and one allele with a 2 bp deletion and one allele with a 1 bp deletion in the HOIP locus. The protospacer-adjacent motif (PAM) sequence is depicted in red.

(B) Immunoblot analysis of lysates from TKO MEFs.

(C and D) GST-mHOIP$_{466–630}$ was immobilized on a SPR sensor chip via a GST antibody. Binding between mHOIP$_{466–630}$ and MBP-mSHARPIN UBL$_{163–301}$ (C) or MBP-mHOIL-1L UBL$_{1–140}$ (D) was evaluated.

(E) Conservation residues in N-terminal regions of UBLs of HOIL-1L and SHARPIN.

(F) Binding affinity between mHOIP$_{466–630}$ and UBLs of mSHARPIN or mHOIL-1L containing or lacking N-terminal regions was analyzed.

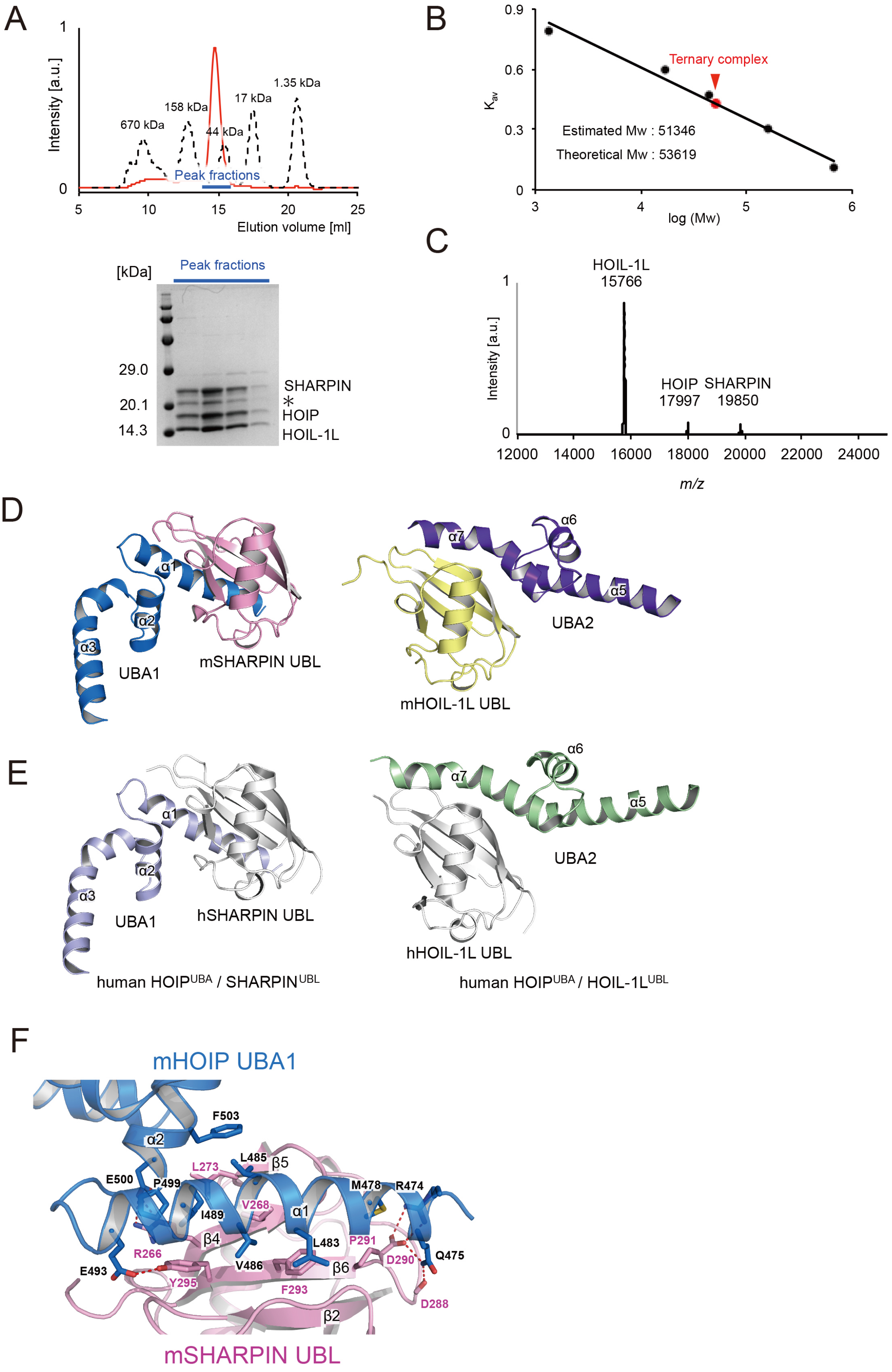

A
Intensity [a.u.]
670 kDa
158 kDa
44 kDa
17 kDa
1.35 kDa
Peak fractions
Elution volume [ml]
[kDa]
Peak fractions
29.0
20.1
14.3
SHARPIN
*
HOIP
HOIL-1L
B
Ternary complex
Estimated Mw : 51346
Theoretical Mw : 53619
log (Mw)
C
HOIL-1L
15766
HOIP 17997
SHARPIN 19850
Intensity [a.u.]
m/z
D
UBA1
mSHARPIN UBL
UBA2
mHOIL-1L UBL
E
UBA1
hSHARPIN UBL
UBA2
hHOIL-1L UBL
human HOIPUBA / SHARPINUBL
human HOIPUBA / HOIL-1LUBL
F
mHOIP UBA1
mSHARPIN UBL
F503
L485
L273
E500
P499
M478
R474
I489
V268
L483
P291
R266
D290
Q475
E493
Y295
V486
F293
D288

Figure S3. Purification of the LUBAC ternary complex core, and comparison of the trimeric core and binary complexes (Related to Figure 3)

(A) Elution profile of size-exclusion chromatography of the LUBAC ternary complex core (red line). The elution profile of standard molecular weight markers is presented as a black dotted line. Protein fractions in the main peak (indicated by a blue line) were analyzed by SDS-PAGE (theoretical molecular weights: HOIP, 17,972; HOIL-1L, 15,744; SHARPIN, 19,903). Asterisk indicates an artifact of SDS-PAGE.

(B) Molecular weight estimation of the LUBAC ternary complex core, based on elution volume in analytical size exclusion column chromatography (A). Partition coefficients (K_{av}) of the trimeric LUBAC core and standard proteins were calculated from their elution volumes. The calibration curve was generated by plotting K_{av} of each standard protein against a logarithm of its molecular weight. The molecular weight of the trimeric LUBAC core was estimated as 51.3 kDa, indicating that the three subunits are assembled in a 1:1:1 stoichiometry (theoretical molecular weight: 53,619).

(C) MS spectra of the protein fraction in (A). The three components co-existed in a single fraction.

(D) Comparison of UBL-binding mode between UBA1 and UBA2 in the ternary complex core. The α1 helix in UBA1 binds the surface of the conserved hydrophobic patch on the β-sheet of the mSHARPIN UBL, whereas α7 helix of UBA2 contacts with the opposite surface of the β-sheet of the mHOIL-1L UBL.

(E) UBA–UBL binding modes observed in the crystal structures of the human HOIP/SHARPIN (left panel, PDB:5X0W) and HOIP/HOIL-1L (right panel, PDB:4DBG) binary complexes. The UBAs are colored as in Figure 3E and 3F, whereas the UBLs are in white.

(F) Interface between mHOIP UBA1 and mSHARPIN UBL. Each subunit is colored as in Figure 3. The interface is mainly formed between helices α1–α2 of mHOIP UBA1 and strands β4–β6 of mSHARPIN UBL. mHOIP and mSHARPIN residues involved in the interaction are shown in stick models. Hydrogen bonds are indicated by red dotted lines.

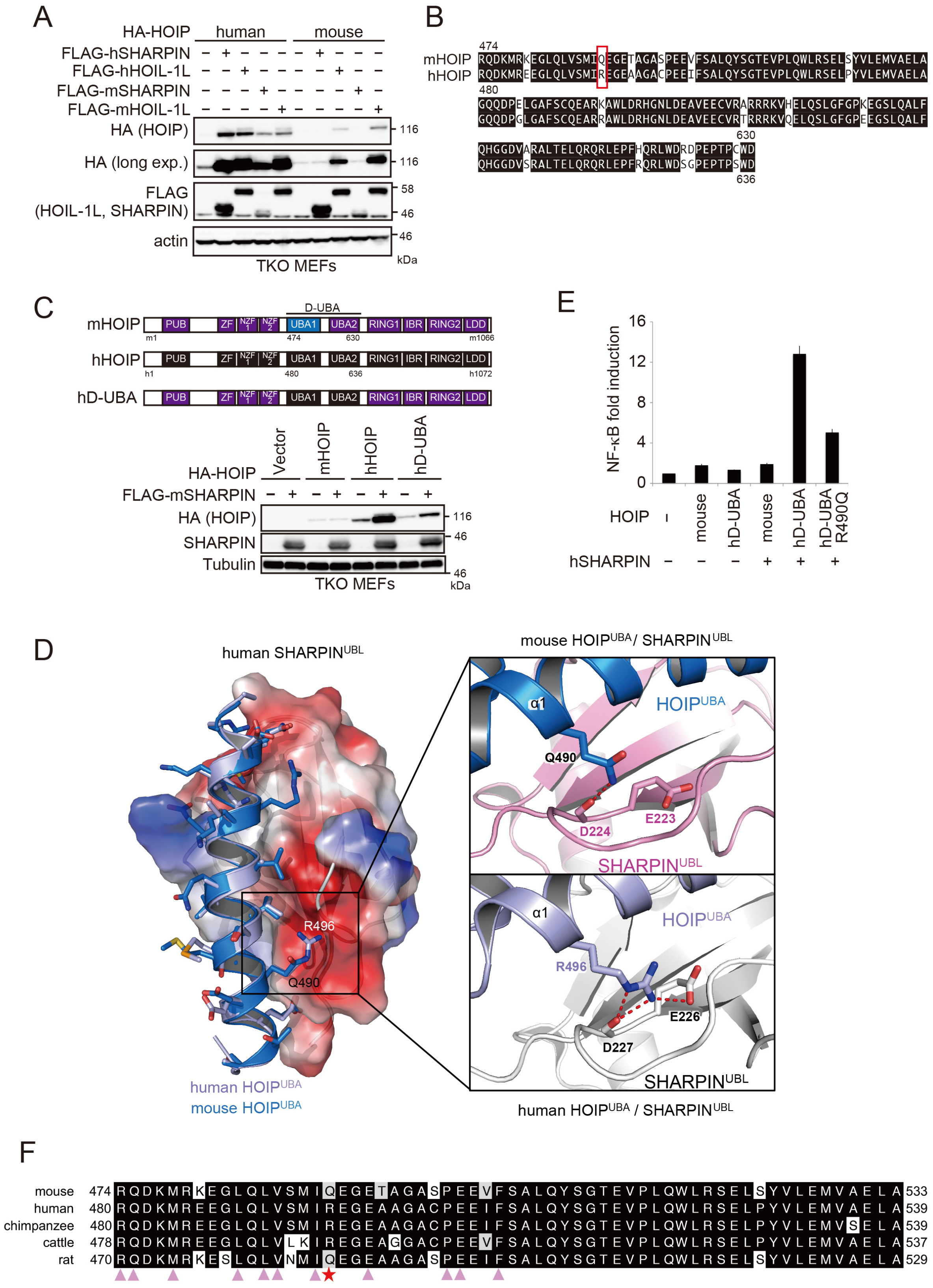
A
HA-HOIP
human
mouse
FLAG-hSHARPIN
FLAG-hHOIL-1L
FLAG-mSHARPIN
FLAG-mHOIL-1L
HA (HOIP)
HA (long exp.)
FLAG
(HOIL-1L, SHARPIN)
actin
TKO MEFs
116
58
46
kDa
B
mHOIP
hHOIP
474
480
630
636
RQDKMRKEGLQLVSMIQEGETAGASPEEVFSALQYSGTEVPLQWLRSELSYVLEMVAELA
RQDKMREEGLQLVSMIREGEAAGACPEEIFSALQYSGTEVPLQWLRSELPYVLEMVAELA
GQQDPELGAFSCQEARKAWLDRHGNLDEAVEECVRARRRKVHELQSLGFGPKEGSLQALF
GQQDPGLGAFSCQEARRAWLDRHGNLDEAVEECVRTRRRKVQELQSLGFGPEEGSLQALF
QHGGDVARALTELQRQRLEPFHQRLWDRDPEPTPCWD
QHGGDVSRALTELQRQRLEPFRQRLWDSGPEPTPSWD
C
D-UBA
mHOIP
hHOIP
hD-UBA
PUB
ZF
NZF 1
NZF 2
UBA1
UBA2
RING1
IBR
RING2
LDD
m1
474
630
m1066
h1
480
636
h1072
HA-HOIP
Vector
mHOIP
hHOIP
hD-UBA
FLAG-mSHARPIN
HA (HOIP)
SHARPIN
Tubulin
TKO MEFs
116
46
46
kDa
E
NF-κB fold induction
0
4
8
12
16
HOIP
mouse
hD-UBA
mouse
hD-UBA
hD-UBA R490Q
hSHARPIN
D
human SHARPIN^UBL
mouse HOIP^UBA / SHARPIN^UBL
R496
Q490
human HOIP^UBA
mouse HOIP^UBA
α1
HOIP^UBA
Q490
D224
E223
SHARPIN^UBL
α1
HOIP^UBA
R496
E226
D227
SHARPIN^UBL
human HOIP^UBA / SHARPIN^UBL
F
mouse 474 RQDKMRKEGLQLVSMIQEGETAGASPEEVFSALQYSGTEVPLQWLRSELSYVLEMVAELA 533
human 480 RQDKMREEGLQLVSMIREGEAAGACPEEIFSALQYSGTEVPLQWLRSELPYVLEMVAELA 539
chimpanzee 480 RQDKMREEGLQLVSMIREGEAAGACPEEIFSALQYSGTEVPLQWLRSELPYVLEMVSELA 539
cattle 478 RQDKMREEGLQLVLKIREGEAGGACPEEVFSALQYSGTEVPLQWLRSELPYVLEMVAELA 537
rat 470 RQDKMRKESLQLVNMIQEGEAAGASPEEIFSALQYSGTEVPLQWLRSELSYVLEMVAELA 529

Figure S4. The difference between mouse and human HOIP UBA1 is critical for SHARPIN-mediated HOIP stabilization (Related to Figures 2 and 3)

(A) Cell lysates of TKO MEFs expressing the indicated proteins were probed with indicated antibodies.

(B) Sequence alignment of human and mouse HOIP D-UBA. Amino acid residues of mHOIP Q490 and the corresponding residue of hHOIP are enclosed in a red box.

(C) Schematic representation of mHOIP, hHOIP, and hD-UBA. Cell lysates of TKO MEFs reconstituted with indicated proteins were probed as indicated.

(D) Structural comparison of the HOIP UBA1/SHARPIN UBL interfaces of mouse and human. Helix $\alpha 1$ of mHOIP UBA is superimposed onto that of hHOIP UBA, which is bound to hSHARPIN UBL. Surface charge (red, negative; blue, positive) of hSHARPIN UBL is shown. Side chains of UBAs are shown in stick models. Close-up views around R496 of hHOIP and Q490 of mHOIP are shown in the right panels. Hydrogen bonds and electrostatic interactions are indicated as red dotted lines.

(E) The indicated expression plasmids and 5× NF-κB luciferase reporter were transfected into HEK293T cells. NF-κB activity was measured by luciferase assays (mean ± s.e.m., $n = 3$).

(F) Conserved residues in HOIP UBA1 of various species.

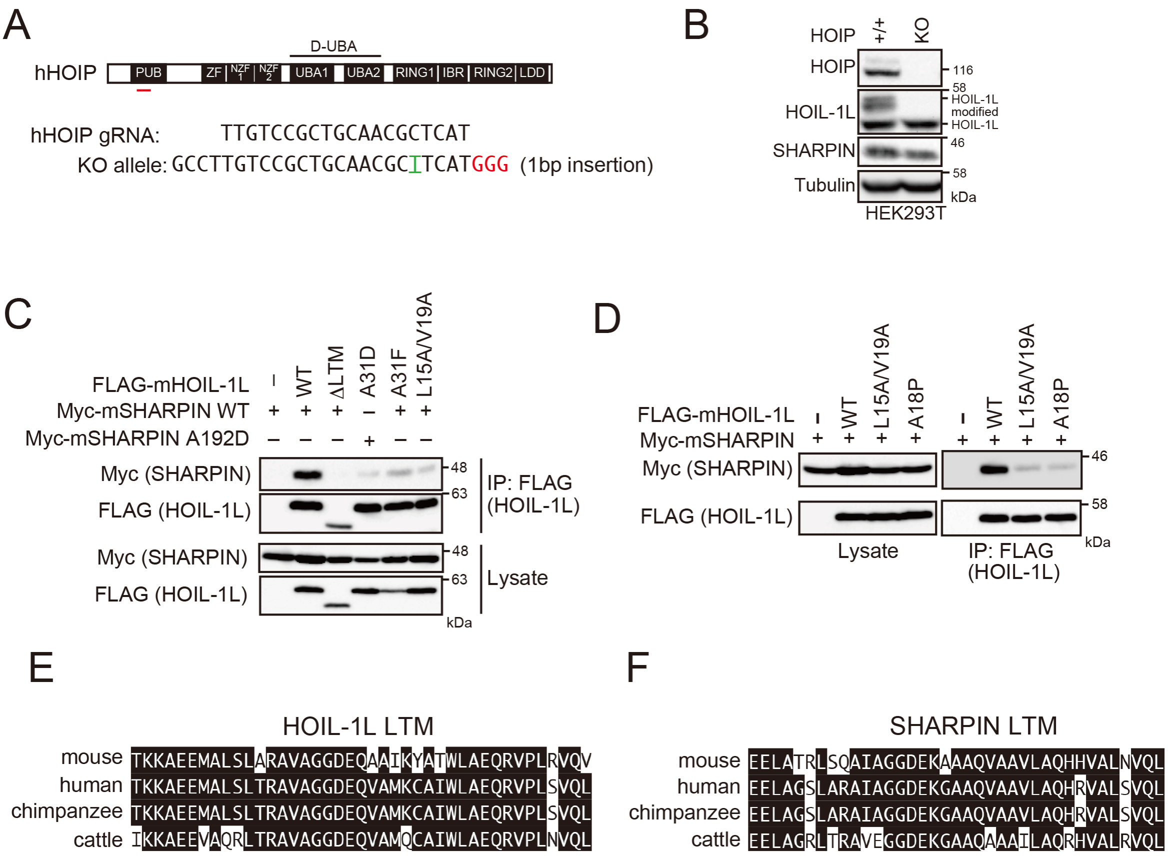

Figure S5. Characterization of LTM mutants (Related to Figures 4 and 5)

(A) Guide RNA sequence against hHOIP. HEK293T HOIP KO cells were homozygous for a 1 bp insertion (depicted in green). The protospacer-adjacent motif (PAM) sequence is depicted in red.

(B) Immunoblot analysis of HEK293T HOIP KO cells. Cell lysates of parent and HEK293T HOIP KO cells were immunoblotted with the indicated antibodies.

(C) The indicated expression plasmids were transfected into HEK293T HOIP KO cells. Cell lysates and anti-FLAG immunoprecipitates were probed as indicated.

(D) The indicated expression plasmids were transfected into HEK293T HOIP KO cells. Cell lysates and anti-FLAG immunoprecipitates were probed as indicated.

(E and F) Conserved residues in the LTM regions of HOIL-1L (E) and SHARPIN (F) from various species.

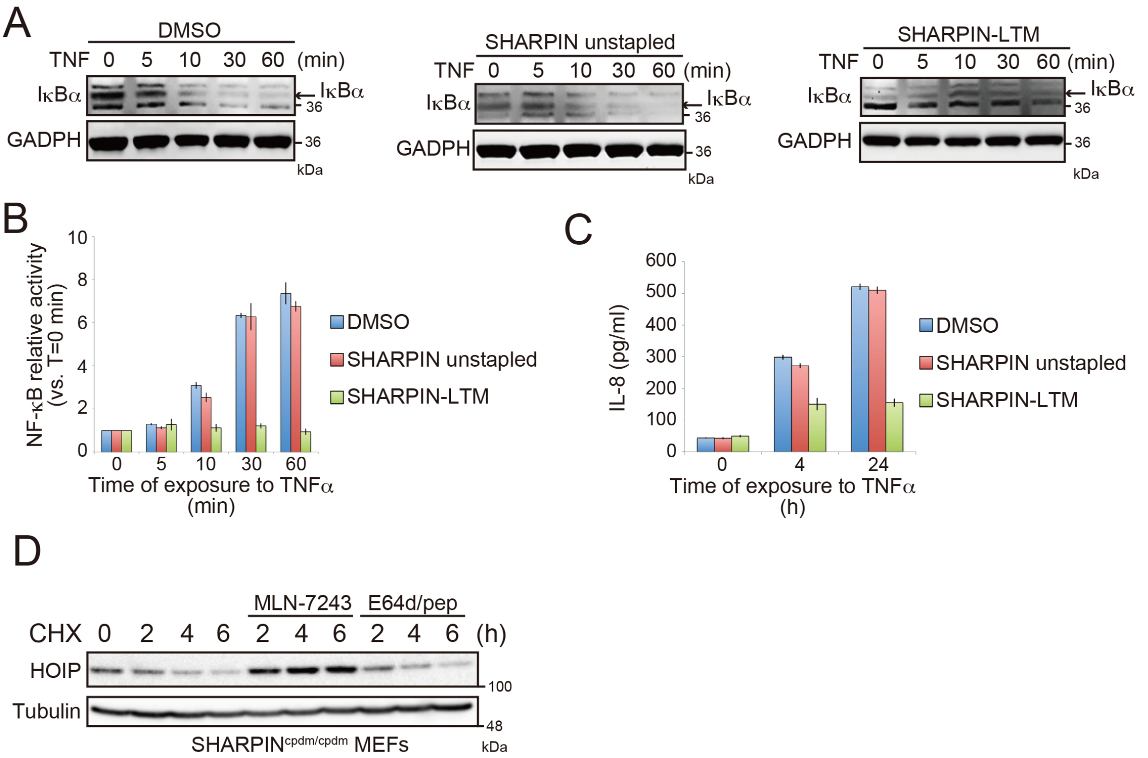

Figure S6. Therapeutic potential of SHARPIN-LTM peptide against ABC-DLBCL cell lines (Related to Figure 6)

(A–C) HBL1 cells were treated for 2 h with 20 μM of each peptide. After incubation, cells were stimulated with TNF-α (5 ng/ml) for the indicated periods. Cell lysates were analyzed for IκBα levels by immunoblotting (A), NF-κB activity by TransAM® NF-κB p65 assay (B), and secreted IL-8 by ELISA (means ± s.e.m., n = 3) (C). (D) Reduction of HOIP by destabilization of LUBAC seems to be mediated by ubiquitin-proteolytic pathway. SHARPIN$^{cpdm/cpdm}$ MEFs were treated with CHX (20 μg/ml) in the presence of MLN-7243 (10 μM) or E64d/pep (10 μg/ml) for the indicated periods. Cell lysates were probed by immunoblotting.

Table S1 Dissociation constants (K_d) from SPR experiments (Related to Figures 4 and S2)

Interactions	K_d
HOIP UBA / SHARPIN UBL	16.0±2.3 μM
HOIP UBA / HOIL-1L UBL	1.34±0.87 μM
HOIL-1L LTM-UBL / SHARPIN LTM-UBL	2.04±0.29 μM

Table S2 Data collection and refinement statistics (Related to Figure 3)

Data collection	
Wavelength (Å)	0.98000
Resolution (Å)	48.18–2.40 (2.44–2.40)
Space group	*C*2
Cell dimensions	
a, *b*, *c* (Å)	143.902, 59.732, 58.554
α, β, γ (°)	90.00, 97.42, 90.00
Total measurements	87,510
Unique cell reflections	19,275
Average redundancy	4.5 (4.2)
I/σ	14.2 (1.9)
Completeness (%)	99.3 (95.5)
R_{merge}	0.092 (0.520)
Refinement	
Resolution (Å)	48.18–2.40
No. reflections	17862
R_{work} (%)	18.8
R_{free} (%)	24.4
No. atoms	3357
Protein	3326
Water	31
Average B-factor (Å^2)	64.8
r.m.s.d.	
Bond length (Å)	0.013
Bond angles (°)	1.692
Ramachandran plot	
Residues in favored region (%)	96.2
Residues in allowed region (%)	3.6
Residues in outlier region (%)	0.2

Values in parentheses correspond to the highest-resolution shell.

Table S3. RESOURCES TABLE

REAGENT or RESOURCE	SOURCE	IDENTIFIER
Antibodies		
Anti-mouse HOIP	Tokunaga et al., 2009	N/A
Anti-human HOIP, 1CB2	Merck Millipore	Cat# MABE1122
Anti-human HOIP	Aviva Systems Biology	Cat# ARP43241_P050
Anti-HOIL-1L, 2E2	Merck Millipore	Cat# AP1140
Anti-HOIL-1L, N-terminal	This study	N/A
Anti-SHARPIN	Merck Millipore	Cat# ABF128
Anti-SHARPIN	Abcam	Cat# ab125188
Anti-phospho IκBα	Cell Signaling Technology	Cat# 9246
Anti-IκBα	Cell Signaling Technology	Cat# 4812
Anti-IκBα, C-21	Santa Cruz Biotechnology	Cat# sc-371
Anti-Caspase3	Cell Signaling Technology	Cat# 9662
Anti-Ubiquitin, P4D1	Santa Cruz Biotechnology	Cat# sc-8017
Anti-β-actin	SIGMA	Cat# A5316
Anti-β-actin	SIGMA	Cat#A2228
Anti-β-tubulin	CEDARLANE	Cat# CLT9002
Anti-GADPH	BIOLEGEND	Cat# 631402
Anti-CD31, clone MEC13.3	BioLegends	Cat# 102502
Anti-HA, Y11	Santa Cruz Biotechnology	Cat# sc-805
Anti-HA, Tana2	MBL	Cat# M180-3
Anti-Myc, 4A6	Merck Millipore	Cat# 05-724
Anti-Myc, 9E10	In house	N/A
Anti-FLAG, M2	SIGMA	Cat# F3165
Anti-FLAG	SIGMA	Cat# F7425
Anti-DDDDK-tag	MBL	Cat# PM020
strepMAB-immo	iba	Cat# 2-1517-001
Bacterial and Virus Strains		
DH5α	N/A	N/A
BL21-CodonPlus(DE3)-RIPL Competent Cells	Agilent Technologies	Cat# 230280
BL21(DE3) Competent Cells	Novagen	Cat# 69450
Rosetta 2(DE3) Competent Cells	Novagen	Cat# 71397
Chemicals, Peptides, and Recombinant Proteins		
Cycloheximide	Calbiochem	Cat# 239674
ATP	GE Healthcare	Cat# 272056
puromycin	SIGMA	Cat# P9620
G-418	NacalaiTesque	Cat# 09380-44
Blasticidin	invivoGen	Cat# ant-bl-1
Dithiothreitol	Nacalai Tesque	Cat# 14112-94
DNaseI	Roche	Cat# 10104159001
Complete, EDTA-free	Roche	Cat# 05056489001

IRDye 700 NF-kB consensus oligonucleotide	LI-COR Biosciences	Cat# 829-07924
MLN-7243	Active Biochem	Cat# A-1384
E-64-d	Peptide Institute	Cat# 4321-v
PepstatinA	Peptide Institute	Cat# 4397-v
HOIP-N peptide	Yang et al., 2014	N/A
SHARPIN unstapled peptide	This study	N/A
SHARPIN-LTM peptide	This study	N/A
Recombinant mouse TNF	R&D systems	Cat# 410-MT
Recombinant human TNF	Promega	Cat# G524A
Recombinant TNF	PEPROTECH	Cat# 300-01A
Recombinant human LUBAC	Tokunaga et al., 2011	N/A
Recombinant E1	Kirisako et al., 2006	N/A
Recombinant UbcH7	Kirisako et al., 2006	N/A
Recombinant Ubiquitin	BostonBiochem	Cat# U-100H
GST-mHOIP UBA (aa 466-630) WT	This study	N/A
GST-mHOIP UBA (aa 466-630) Q607A/L611A/F614A	This study	N/A
GST-mHOIP UBA (aa 466-630) R474A/L483A/V486A	This study	N/A
MBP-mHOIL-1L UBL (aa 1-140) WT	This study	N/A
MBP-mHOIL-1L UBL (aa 1-140) L15A/V19A	This study	N/A
MBP-mHOIL-1L UBL (aa 37-161)	This study	N/A
MBP-mSHARPIN UBL (aa 163-301) WT	This study	N/A
MBP-mSHARPIN UBL (aa 163-301) L176A/I180A	This study	N/A
MBP-mSHARPIN UBL (aa 198-318)	This study	N/A
mHOIL-1L UBL (aa 1-189)-strep	This study	N/A
His-mHOIP (aa 474-630)	This study	N/A
mHOIL-1L (aa 1-140)	This study	N/A
GST-SHARPIN (aa 163-341)	This study	N/A
ProTEV Plus	Promega	Cat# V6101
Recombinant HRV3C	This study	N/A
Gel Filtration Standard	Bio-Rad	Cat# 151-1901
MES monohydrate	Hampton Research	Cat# HR2-243
Magnesium sulfate hydrate	Hampton Research	Cat# HR2-633
Glycerol	Nacalai Tesque	Cat# 17018-83
Critical Commercial Assays		
GST-Capture Kit	GE Healthcare	Cat# BR-1002-23
Mouse antibody capture kit	GE Healthcare	Cat#
Dual-Luciferase Reporter Assay System	Promega	Cat# E1980
High Capacity RNA to cDNA kit	ThermoFisher	Cat# 4387406
Topo cloning Kit	invitrogen	Cat# 45-0641
E-Plate L8 PET	ACEA Biosiences	Cat# 00300500870
In Situ Cell Death Detection Kit, Fluorescein	Roche	Cat# 11684795910
Odyssey Infrared EMSA kit	LI-COR Biosciences	Cat# 829-07910
TransAM® p65	ACTIVEMOTIF	Cat# 40096
IL-8 ELISA	PEPROTECH	Cat#900-T18
CellTiter-Glo Luminescent Cell Viability Assay kit	Promega	Cat# G7570

Deposited Data		
Atomic coordinates, mouse LUBAC ternary complex core	This study	PDB: 5Y3T
Experimental Models: Cell Lines		
HEK293T	N/A	N/A
HEK293T HOIP KO	This study	N/A
Jurkat	N/A	N/A
Jurkat HOIP KO	Sakamoto et al., 2015	N/A
Immortalized MEF HOIL-1L$^{-/-}$	Tokunaga et al., 2009	N/A
Immortalized MEF HOIL-1L$^{+/+}$	Tokunaga et al., 2009	N/A
Immortalized MEF HOIL-1L$^{null/null}$	This study	N/A
Immortalized MEF HOIL-1L$^{+/+}$	This study	N/A
Immortalized MEF HOIP$^{\Delta linear/\Delta linear}$	Shimizu et al., 2016	N/A
Immortalized MEF SHARPIN$^{cpdm/cpdm}$	Tokunaga et al., 2011	N/A
LUBAC TKO MEF	This study	N/A
HBL1	N/A	N/A
Experimental Models: Organisms/Strains		
Mouse: HOIL-1L^{null}	This study	N/A
Mouse: HOIL-1L$^{null\#2}$	This study	N/A
Oligonucleotides		
Mouse HOIL-1L primer: Exon4_Fwd, 5’-GGAATGGAGACGGTGCCTATCTC-3’	This study	N/A
Mouse HOIL-1L primer: Exon5_Fwd, 5’-CGAAGCCCAGGACCAACCAGGAG-3’	This study	N/A
Mouse HOIL-1L primer: Exon6_Fwd, 5’-TGTGAGATGTGCTGTCGTGCAAG-3’	This study	N/A
Mouse HOIL-1L primer: Exon8_Fwd, 5’-CATTGACAGCACCTACTCATGCC-3’	This study	N/A
Mouse HOIL-1L primer: Exon5_Rev, 5’-CTGGGCTTCGGAAGGACAGGTTC-3’	This study	N/A
Mouse HOIL-1L primer: Exon9_Rev, 5’-CTCAAGGTGCTTCGGTTCTCTG-3’	This study	N/A
Mouse HOIL-1L primer: Exon10_Rev, 5’-TCCCTGCAATTCATGTGCTCATG-3’	This study	N/A
Mouse β-actin primer: Fwd, 5’-ATGGATGACGATATCGCTC-3’;	This study	N/A
Mouse β-actin primer: Rev, 5’-GATTCCATACCCAGGAAGG-3’	This study	N/A
mHOIL-1L typing primer: Fwd, 5’-TTGCCAACAGGCCAATTTGATG-3’	This study	N/A
mHOIL-1L typing primer Rev, 5’-TGCGGTGATGCACAATATCCTG-3’	This study	N/A
mHOIP typing primer Fwd, 5’-AGCGCCCTGAGGTGGGATT-3’	This study	N/A
mHOIP typing primer Rev, 5’-GCGCTCCTCAGTATAGCCATACAACC-3’	This study	N/A
hHOIP typing primer Fwd, 5’-TTCCGGGCAGGCGTTTTCCCTG-3’	This study	N/A
hHOIP typing primer Rev, 5’-CTCTGTGTAGCCATATAATCG-3’	This study	N/A
Recombinant DNA		

pcDNA3.1 HA-mHOIP WT	Fujita et al., 2014	N/A
pcDNA3.1 HA-hHOIP	Kirisako et al., 2006	N/A
pcDNA3.1 HA-hD-UBA	This study	N/A
pcDNA3.1 HA-hD-UBA R490Q	This study	N/A
pcDNA3.1 FLAG-mHOIL-1L WT	Fujita et al., 2014	N/A
pcDNA3.1 Myc-mHOIL-1L WT	Fujita et al., 2014	N/A
pcDNA3.1 FLAG-mHOIL-1L Δ_{aa}1-36	This study	N/A
pcDNA3.1 FLAG-mHOIL-1L L15A	This study	N/A
pcDNA3.1 FLAG-mHOIL-1L V19A	This study	N/A
pcDNA3.1 FLAG-mHOIL-1L L15A/V19A	This study	N/A
pcDNA3.1 FLAG-mHOIL-1L A31D	This study	N/A
pcDNA3.1 FLAG-mHOIL-1L A31F	This study	N/A
pcDNA3.1 FLAG-mHOIL-1L A18P	This study	N/A
pcDNA3.1 FLAG-hHOIL-1L WT	This study	N/A
pcDNA3.1 FLAG-hHOIL-1L A18P	This study	N/A
pcDNA3.1 FLAG-mSHARPIN WT	Fujita et al., 2014	N/A
pcDNA3.1 FLAG-hSHARPIN WT	Tokunaga et al., 2011	N/A
pcDNA3.1 Myc-mSHARPIN WT	Fujita et al., 2014	N/A
pcDNA3.1 FLAG-mSHARPIN Δ_{aa}163-197	This study	N/A
pcDNA3.1 Myc-mSHARPIN Δ_{aa}163-197	This study	N/A
pcDNA3.1 FLAG-mSHARPIN L176A	This study	N/A
pcDNA3.1 FLAG-mSHARPIN I180A	This study	N/A
pcDNA3.1 FLAG-mSHARPIN L176A/I180A	This study	N/A
pcDNA3.1 Myc-mSHARPIN L176A/I180A	This study	N/A
pcDNA3.1 Myc-mSHARPIN A192D	This study	N/A
pcDNA3.1 Myc-mS(UBL)-HOIL-1L	This study	N/A
pcDNA3.1 Myc-mH(UBL)-SHARPIN	This study	N/A
pMXs-neo-HA-mHOIP	This study	N/A
pMXs-neo-HA-hHOIP	This study	N/A
pMXs-neo-HA-hD-UBA	This study	N/A
pMXs-IP-FLAG-mHOIL-1L WT	This study	N/A
pMXs-IP-FLAG-mHOIL-1L UBL	This study	N/A
pMXs-IP-FLAG-hHOIL-1L WT	This study	N/A
pMXs-IP-FLAG-mSHARPIN WT	This study	N/A
pMXs-IP-FLAG-hSHARPIN WT	This study	N/A
pMXs-IRES-Bsr	This study	N/A
pMXs-IRES-Bsr-mSHARPIN	This study	N/A
pMXs-puro-IRES-att-FLAG-mSHARPIN WT	This study	N/A
pMXs-puro-IRES-att-FLAG-mSHARPIN L176A/I180A	This study	N/A
pcDNA3.1-MMTV FLAG	Tokunaga et al., 2009	N/A
pcDNA3.1-MMTV FLAG mHOIL-1L WT	This study	N/A
pcDNA3.1-MMTV FLAG mHOIL-1L A18P	This study	N/A
pGEX 6p1 mHOIP D-UBA (aa 466-630) WT	This study	N/A
pGEX 6p1 mHOIP D-UBA Q607A/L611A/F614A	This study	N/A
pGEX 6p1 mHOIP D-UBA R474A/L483A/V496A	This study	N/A
pMAL c2x mHOIL-1L (aa 1-140) WT	This study	N/A
pMAL c2x mHOIL-1L (aa 1-140) L15A/V19A	This study	N/A
pMAL c2x mHOIL-1L (aa 37-161) WT	This study	N/A

pMAL c2x mSHARPIN (aa 163-301) WT	This study	N/A
pMAL c2x mSHARPIN (aa 163-301) L176A/I180A	This study	N/A
pMAL c2x mSHARPIN (aa 198-318) WT	This study	N/A
pT7-7 mHOIL-1L (aa 1-189)–strep WT	This study	N/A
pETDuet-1 mHOIP (aa 474-630) WT, mHOIL-1L (1-140)	This study	N/A
pGEX 6p1 mSHARPIN (163-341)	This study	N/A
pGEX 6p1 HRV3C	This study	N/A
pGL4.32[luc2P/NF-κB-RE/Hygro]	Promega	Cat# E8491
pGL4.74[hRluc/TK]	Promega	Cat# E6921
px330	Addgene	Cong et al., 2013
px459	Addgene	Ran et al., 2013
Software and Algorithms		
CRISPR design	http://crispr.mit.edu	N/A
PyMol	PyMOL	http://www.pymol.org
Image J	https://imagej.nih.gov/ij/	N/A
PRISM ver.5	PRISM ver.5	http://www.graphpad.com
HKL2000	Otwinowski and Minor, 1997	http://www.hkl-xray.com/
CCP4 package	Winn et al., 2011	www.ccp4.ac.uk
DSSP	Touw et al., 2015	http://www.cmbi.ru.nl/xssp/
COOT	Emsley et al., 2010	https://www2.mrc-lmb.cam.ac.uk/personal/pemsley/coot/
Other		
Ni-NTA Agarose	Qiagen	Cat# 30230
Glutathione Sepharose 4B	GE Healthcare	Cat# 17-0756-05
HiTrap Q HP	GE Healthcare	Cat# 17-1154-01
HiLoad 16/600 Superdex 200 pg	GE Healthcare	Cat# 28-9893-35
Superdex 200 10/300 GL	GE Healthcare	Cat# 17-5175-01
Amylose Resin	NEB	Cat# E8021
rmp proteinA Sepharose Fast Flow	GE Healthcare	Cat# 17-5138-02
rProtein A Sepharose Fast Flow	GE Healthcare	Cat# 17-1270-02
Glutathione Sepharose 4 Fast Flow	GE Healthcare	Cat# 17-5132-02
Strep-Tactin Sepharose	IBA	Cat# 2-1201-010

SUPPLEMENATL EXPERIMENTAL PROCEDURES

Plasmids

cDNAs used in this study were described previously (Fujita et al., 2014; Tokunaga et al., 2011; Tokunaga et al., 2009). The hD-UBA and hD-UBA R490Q [mHOIP (aa 1–473)–hHOIP (aa 480–636)–mHOIP (aa 631–1066)] were generated from the amplified ORFs of human and mouse HOIP. The following proteins were generated from the amplified ORF of mouse HOIL-1L: before NZF (aa 1–189), UBL (aa 1–140), Δ_{aa}1–36 (aa 37–509), and UBL Δ_{aa}1–36 (aa 37–161). The following proteins were generated from the amplified ORF of mouse SHARPIN: Δ_{aa}163–197 (Δaa 163–197), UBL (aa 163–301 or 163–340), and UBL Δ_{aa}163–197 (aa 198–318). The following mutants of mHOIL-1L and mSHARPIN, in which the UBLs were exchanged, were generated from the amplified ORFs of mouse HOIL-1L and SHARPIN: S(UBL)-HOIL [SHARPIN (aa 163–301)–HOIL-1L (aa 136–509)] and H(UBL)-SHARPIN [SHARPIN (aa 1–167)–HOIL-1L (aa 7–135)–SHARPIN (aa 302–380)]. Mutants of mHOIP (R474A/L483A/V486A, Q607A/L611A/F614A), mHOIL-1L (L15A, A18P, V19A, A31D, A31F, L15A/V19A), mSHARPIN (L176A, I180A, A192D, L176A/I180A), and hHOIL-1L (A18P) were generated by two-step PCR. cDNAs were ligated into the appropriate epitope-tag sequences, and then cloned into pcDNA3.1, pDNA3.1-MMTV, pMAL-c2x, pGEX-6p1, pMXs-IP, pMXs-neo, pMXs-puro-IRES-att (Bochkov and Palmenberg, 2006), and pMXs-IRES-Bsr. pX330-U6-Chimeric_BB-CBh-hSpCas9 (Addgene plasmid #42230) (Cong et al., 2013) and pSpCas9(BB)-2A-Puro (PX459) (Addgene plasmid #48139) (Ran et al., 2013) were obtained from Addgene.

Antibodies and reagents

Antibodies against the following proteins were purchased from Cell Signaling Technology and used at the indicated dilutions for western blot analysis: p-IκBα (9246, 1:2000), IκBα (4812, 1:2000), and caspase-3 (9662, 1:2000). Antibodies against ubiquitin (P4D1) (sc-8017, 1:2000) and IκBα (C-21) (sc-371, 1:2000) were purchased from Santa Cruz Biotechnology. Antibodies against β-actin (A5316, 1:10,000), β-actin (A2228, 1:10,000), and FLAG (M2) (F3165, 1:2000) used for western blot analysis, and anti-FLAG antibody (F7425, 1:150) used for immunoprecipitation, were purchased from Sigma. Anti-GAPDH (631402, 1:2000) used for western blot analysis, and anti-CD31 (MEC13.3) (102502, 1:100) used for immunohistochemistry, were purchased from BioLegend. Antibodies against HA (Tana2) (M180-3, 1:2000) and DDDDK-tag (PM020, 1:2000) were purchased from MBL and used for western blot analysis. Anti-Myc (4A6) (05-724, 1:2000) was purchased from Merck Millipore, anti–β-tubulin (CLT9002, 1:10,000) was purchased from CEDARLANE, anti-HOIP (ARP43241_P050, 1:2000) was purchased from Aviva Systems Biology, anti-SHARPIN (ab125188, 1:2000) was purchased from Abcam, and StrepMAB-Immo (2-1517-001) was purchased from IBA. Antibodies against the following proteins were made in-house (Fujita et al., 2014; Tokunaga et al., 2011; Tokunaga et al., 2009): mouse HOIP (1:2000), human HOIP (1CB2) (1:2000), HOIL-1L (2E2) (1:2000), and SHARPIN (1:2000). To generate the anti-mouse HOIL-1L N-term antibody, strep-tagged mouse HOIL-1L N-terminus (aa 1–189) was expressed in *Escherichia coli* (Agilent Technologies), and then purified using Strep-Tactin Sepharose (IBA). Purified protein was used to immunize rabbits, and IgG was purified from antisera using Protein A–Sepharose

(GE Healthcare). SHARPIN peptide synthesis, reverse-phase high-performance liquid chromatography purification, and amino acid analysis were performed as described previously (Bernal et al., 2007).

Cell lines

HEK293T cells, HEK293T HOIP KO cells, MEFs derived from WT, cpdm, HOIP Δlinear, HOIL-1L$^{-/-}$, or HOIL-1L–null mice, and LUBAC TKO MEFs were grown in Dulbecco's modified Eagle's medium (DMEM) plus 10% fetal bovine serum (FBS) with 100 IU/ml penicillin and 100 μg/ml streptomycin, at 37°C under 7.5% CO_2. HBL1 cells were grown at 37°C under 7.5% CO_2 in RPMI 1640 medium supplemented with 10% FBS, 100 IU/ml penicillin, and 100 μg/ml streptomycin.

Transfection and retroviral expression

Transfections were performed using Lipofectamine 2000. For retroviral expression, pMXs-IP, pMXs-puro-IRES-att, pMXs-neo, or pMXs-IRES-Bsr containing LUBAC components were transfected into Plat E packaging cells. The resultant viruses were used to infect LUBAC TKO MEFs, cpdm MEFs or HOIL-1L–null MEFs, and stably transduced cells were selected using puromycin, G-418, or blasticidin.

Cell lysis and immunoprecipitation

Cells were lysed with lysis buffer [50 mM Tris-HCl (pH 7.5), 150 mM NaCl, 1% Triton X-100, 2 mM PMSF, and protease inhibitor cocktail (Sigma-Aldrich)], and the lysates were clarified by centrifugation at 15,000 rpm for 20 min at 4°C. For immunoprecipitation, lysates were incubated with the appropriate antibodies for 90 min on ice, and then immobilized on rmp–Protein A–Sepharose beads (GE Healthcare). The beads were washed five times with lysis buffer.

Immunoblotting and EMSA

Samples were separated by SDS-PAGE, and then transferred to PVDF membranes. After blocking in Tris-buffered saline (TBS) containing 0.1% Tween-20/5% (w/v) nonfat dry milk, the membrane was incubated with the appropriate primary antibodies, followed by the corresponding secondary antibodies. The membranes were visualized by enhanced chemiluminescence and analyzed on a LAS4000mini or LAS3000 instrument (GE Healthcare). EMSAs for NF-κB activity were performed using the Odyssey Infrared EMSA kit (LI-COR Biosciences) and IRDye 700 NF-κB consensus oligonucleotide (LI-COR Biosciences), and visualized on an Odyssey 9120 Infrared Imaging System (LI-COR Biosciences).

Purification of the LUBAC ternary complex

cDNA fragments encoding mouse HOIP D-UBA (aa 474–630) and mouse HOIL-1L LTM-UBL (aa 1–140) were inserted between the *EcoR*I and *Not*I sites and the *Nde*I and *EcoR*V sites of pET Duet-1 (Novagen), respectively. A Tobacco Etch Virus (TEV) protease cleavage site was inserted between the N-terminal hexa-histidine tag (His tag) and HOIP D-UBA. A cDNA fragment of mouse SHARPIN LTM-UBL (aa 163–341) was inserted between the *EcoR*I and *Not*I sites of pGEX-6p-1 (GE Healthcare), yielding recombinant mSHARPIN LTM-UBL with extra amino acid residues (LGSPEF) at the N-terminus after GST-tag cleavage with HRV3C protease. The

binary complex of mHOIP D-UBA and mHOIL-1L LTM-UBL was co-expressed in *E. coli* strain BL21 (DE3). Cells were grown at 37°C until OD_{660} reached 0.6; protein expression was induced by addition of IPTG to a final concentration of 0.5 mM, and culture was continued at 18°C overnight. Cells were harvested by centrifugation. The cell pellet was resuspended in Buffer A (50 mM Tris-HCl pH 8.0, 150 mM NaCl, 10% Glycerol, and 1 mM TCEP) and lysed by sonication. The lysate was clarified by centrifugation, and applied to a Ni-NTA agarose column (Qiagen). After extensive column washing, bound proteins were eluted in Buffer A containing 400 mM imidazole. Fractions containing the binary complex were pooled and co-purified with mSHARPIN LTM-UBL as described below. GST-mSHARPIN LTM-UBL was overexpressed in *E. coli* strain Rosetta2 (DE3) (Novagen) in LB medium. Cells were cultured and processed in the same manner as for purification of the HOIP–HOIL-1L complex. The clarified lysate containing GST-mSHARPIN LTM-UBL was applied to a glutathione–Sepharose affinity column (GE Healthcare), and subsequently His-tagged mHOIP D-UBA–mHOIL-1L LTM-UBL complex was added to form the ternary complex. After incubation at 4°C for 30 min, unbound proteins were removed by extensive column washing. The ternary complex was eluted from the column by cleavage of the GST tag with 400 μM HRV3C protease. To cleave the His tag from mHOIP D-UBA, TEV protease (Promega) was added at a final concentration of 250 μM. The ternary complex was applied to a HiTrap Q HP anion exchange column (GE Healthcare) and eluted with a linear NaCl gradient from 50 to 600 mM. The complex was further purified by size-exclusion chromatography using HiLoad Superdex 16/60 200 pg (GE Healthcare) in a buffer containing 50 mM Tris-HCl pH 8.0, 150 mM NaCl, and 1 mM TCEP. Peak fractions containing the stoichiometric ternary complex were combined, concentrated (typically to 7.0 mg/ml), and stored at 4°C until further use.

Analytical size-exclusion chromatography

Analytical size-exclusion chromatography for the LUBAC ternary complex core was performed at 4°C using Superdex 200 10/300 GL (GE Healthcare) in a buffer containing 50 mM Tris-HCl pH 8.0, 150 mM NaCl, and 1 mM TCEP. Fractions were analyzed by SDS-PAGE, and visualized by CBB and mass spectroscopy.

SPR analysis

GST-HOIP UBA (aa 466–630) WT, UBA2^{mut} (Q607A/L611A/F614A), UBA1^{mut} (R474A/L483A/V486A), MBP-HOIL-1L UBL (aa 1–140, 37–161), MBP-HOIL-1L UBL L15A/V19A (aa 1–140), MBP-SHARPIN UBL (aa 163–301, 198–318) and MBP-SHARPIN UBL L176A/I180A (aa 163–301) were expressed in *E. coli* (Agilent Technologies). Expression of GST-HOIP UBA was induced by addition of 0.2 mM IPTG, and culture was continued at 15°C overnight. Expression of MBP-HOIL-1L UBL or SHARPIN UBL was induced by 0.3 mM IPTG, followed by a further 6 h incubation at 25°C. Cells were collected by centrifugation and frozen rapidly. Subsequently, the cells were resuspended in buffer containing 50 mM Tris-HCl (pH 7.5), 150 mM NaCl, 1 mM DTT (Nacalai Tesque), 200 μg/ml lysozyme chloride (Nacalai Tesque), 10 μg/ml DNase (Roche), 2 mM PMSF, and protease inhibitor cocktail (Roche) at 4°C for 30 min, and then lysed in the presence of 0.2% Triton X-100 at 4°C for 20 min. Insoluble material was removed by centrifugation at 14,000 rpm for 20 min at 4°C. GST-HOIP UBA was purified from the supernatant using glutathione–Sepharose beads (GE Healthcare), and MBP-HOIL-1L UBL and MBP-SHARPIN UBL were purified using Amylose Resin (NEB). Binding affinities

between UBA and the UBLs were measured on a Biacore 3000 (GE Healthcare). GST-HOIP UBA was immobilized on a CM5 sensor chip with an anti-GST antibody using the GST capture kit (GE Healthcare) in 10 mM HEPES buffer (pH 7.4) containing 150 mM NaCl and 0.05% (v/v) surfactant P20 at 25°C. Binding between GST-HOIP UBA and MBP-UBLs was measured in 10 mM HEPES buffer (pH 7.4) containing 150 mM NaCl and 0.05% (v/v) surfactant P20 at 25°C. The K_d of HOIL-1L UBL–HOIP UBA or SHARPIN UBL–HOIP UBA was calculated by steady-state affinity analysis. In Figures 2G and 2I, 4E–4G, and S2F, the binding affinities of HOIP UBA for HOIL-1L UBL alone (500 μg/ml), SHARPIN UBL alone (500 μg/ml), and the mixture of HOIL-1L and SHARPIN UBL (500 μg/ml each) were measured. In Figure 4C and 4D, to immobilize HOIL-1L (aa 1–189)-strep, anti-mouse antibody was first immobilized on the sensor chip using the mouse antibody capture kit (GE Healthcare). StrepMAB-Immo (IBA Lifesciences) was then applied to capture HOIL-1L (aa 1–189)-strep. Binding between HOIL-1L (aa 1–189)-strep and MBP-SHARPIN UBLs was measured in 10 mM HEPES buffer (pH 7.4) containing 150 mM NaCl and 0.05% (v/v) surfactant P20 at 25°C.

Generation of cell lines

To generate HOIP–HOIL-1L–SHARPIN TKO MEFs, MEFs from mice with cpdm were electroporated using a NEPA21 electroporator (NEPAGENE) with pX330 plasmid containing a gRNA sequence against mHOIP or mHOIL-1L. After 4 days of culture, cells were seeded at low density. Colonies were picked, and the expression level of LUBAC was analyzed by immunoblotting as a primary screen. Next, genomic regions of HOIL-1L or HOIP were amplified by PCR using the following primers: mHOIL-1L typing_Fwd (5’-TTGCCAACAGGCCAATTTGATG-3’) and typing_Rev (5’-TGCGGTGATGCACAATATCCTG-3’); mHOIP typing_Fwd (5’-AGCGCCCTGAGGTGGGATT-3’) and typing_Rev (5’-GCGCTCCTCAGTATAGCCATACAACC-3’). PCR products were cloned into the TOPO cloning vector (Invitrogen), and mutations were identified by sequencing. To generate HEK293T HOIP KO cells, HEK293T cells were transfected using Lipofectamine 2000 (Invitrogen) with a pX459 plasmid containing a gRNA sequence against hHOIP. Starting on the following day, cells were selected with puromycin for 2 days. Following selection, the cells were seeded at a low density, and isolated colonies were picked. To identify HOIP KO cells, the expression level of HOIP was analyzed by immunoblotting, and the HOIP locus was amplified by PCR using the following primers: hHOIP typing_Fwd (5’-TTCCGGGCAGGCGTTTTCCCTG-3’) and typing_Rev (5’-CTCTGTGTAGCCATATAATCG-3’) PCR products were analyzed by sequencing. Primary MEFs from HOIL-1L–null or WT littermate mice were immortalized with SV-40 large T antigen. In Figure 5F, HOIL-1L$^{null/null}$ MEFs were transfected with pcDNA3.1 MMTV-FLAG mHOIL-1L or A18P using Lipofectamine 2000 (Invitrogen). The next day, cells were seeded at a low density and selected with G-418, and single colonies were picked.

Luciferase assay

HEK293T cells were transfected with pGL4.32 (Luc2p/NF-κB–RE/Hygro) and pGL4.74 (hRLuc/TK) (Promega) along with WT or mutant LUBAC components. At 21–24 h after transfection, cells were lysed, and luciferase activity was measured using the Dual-Luciferase reporter assay system (Promega) on a Lumat Luminometer (Berthold).

Cell viability assay

Cell viability was continuously monitored as an impedance-based cell index using the iCELLigence system (ACEA Bioscience). For each sample, 20,000 cells were plated onto an E-Plate L8 PET (ACEA Bioscience). The next day, cells were treated with TNF-α (10 ng/ml), and cell index was monitored continuously. Plots show data normalized against cell indices at the time of TNF-α treatment. Viability of HBL1 cells in Figure 6H was evaluated using the CellTiter-Glo Luminescent Cell Viability Assay kit (Promega).

Immunohistochemistry

For whole-mount immunohistochemical analyses of CD31, embryos at E10.5 were fixed for 2–4 h with 4% paraformaldehyde in PBS at 4°C, followed by three 30 min washes with PBS-T (PBS with 0.2% Triton X) at 4°C. Next, samples were blocked in 1% BSA in PBS for 1 h at room temperature, and then incubated for 2 days at 4°C with anti-CD31 primary antibody diluted 1:100 in blocking buffer. Samples were washed three times with PBS-T for 30 min at 4°C, followed by two 30 min washes at room temperature, and then incubated overnight at 4°C with Alexa Fluor 546–conjugated secondary antibody diluted 1:1000 in blocking buffer. After five 30 min washes with PBS-T at room temperature, samples were dehydrated with a graded concentration of methanol, and then incubated overnight at 4°C in a graded concentration of methyl salicylate and benzyl alcohol/benzyl benzoate to achieve optimal optical clearing. TUNEL assays were performed using the In Situ Cell Death Detection Kit (Roche) as described previously (Shimizu et al., 2016). Samples were analyzed under a stereoscopic fluorescence microscope.

Ubiquitin and kinase assay

Recombinant His_6-HOIP/HOIL-1L/SHARPIN, E1, and UbcH7 were prepared as described previously (Kirisako et al., 2006; Tokunaga et al., 2011). Trimeric LUBAC (0.2 μM) was incubated on ice for 3 h with DMSO, HOIP-N (80 μM), SHARPIN unstapled (80 μM), and SHARPIN-LTM (80 μM) peptides in buffer containing 20 mM Tris Tris-HCl (pH 7.5) and 1 mM DTT. Next, a mixture of E1 (100 ng), UbcH7 (400 ng), and Ub (5 μg) with or without 2 mM ATP was added [in buffer containing 20 mM Tris Tris-HCl (pH 7.5), 5 mM $MgCl_2$, and 1 mM DTT], and the sample was incubated at 37°C for 30 min.

To prepare S100 lysates (Sakamoto et al., 2015), Jurkat HOIP KO cells were lysed with buffer containing 10 mM Tris-HCl (pH 7.5), 10 mM KCl, 1.5 mM $MgCl_2$, 0.5 mM DTT, 2 mM PMSF, 50 μg/ml leupeptin, and 10 μg/ml aprotinin. Lysates were centrifuged at 15,000 rpm at 4°C for 15 min, and 0.11 volume of buffer containing 0.3 mM Tris-HCl (pH 7.5), 1.4 M KCl, and 30 mM $MgCl_2$ was added to the supernatant. The sample was then centrifuged at 100,000 g at 4°C for 1 h. The resultant supernatant was dialyzed against buffer containing 20 mM Tris-HCl (pH 7.2) and 1 mM DTT. S100 lysates of Jurkat HOIP KO cells (10 μg) and trimeric LUBAC (0.1 μM) were incubated on ice for 3 h with DMSO, HOIP-N (80 μM), SHARPIN unstapled (80 μM), and SHARPIN-LTM (80 μM) peptides in buffer containing 20 mM Tris Tris-HCl (pH 7.5), 1 mM DTT, and 10 μg/ml leupeptin. Next, a mixture of E1 (100 ng), UbcH7 (400 ng), Ub (5 μg), and creatine phosphokinase (Sigma-Aldrich) in buffer containing 20 mM Tris-HCl (pH 7.5), 10 mM $MgCl_2$, 1 mM DTT, 10

mM creatine phosphate, and phosphatase inhibitor cocktail (Nacalai Tesque) with or without 2 mM ATP was added, and the sample was incubated at 37°C for 30 min.

RT-PCR

RNA was isolated from HOIL-1L$^{-/-}$ or WT MEFs using the RNeasy Mini Kit (QIAGEN) and reverse transcribed using the high-capacity RNA-to-cDNA Kit (Applied Biosystems). RT-PCR of HOIL-1L was performed using the following primers:

Exon4_Fwd, 5'-GGAATGGAGACGGTGCCTATCTC-3';
Exon5_Fwd, 5'-CGAAGCCCAGGACCAACCAGGAG-3';
Exon6_Fwd, 5'-TGTGAGATGTGCTGTCGTGCAAG-3';
Exon8_Fwd, 5'-CATTGACAGCACCTACTCATGCC-3';
β-actin_Fwd, 5'-ATGGATGACGATATCGCTC-3';
Exon5_Rev, 5'-CTGGGCTTCGGAAGGACAGGTTC-3';
Exon9_Rev, 5'-CTCAAGGTGCTTCGGTTCTCTG-3';
Exon10_Rev, 5'-TCCCTGCAATTCATGTGCTCATG-3';
β-actin_Rev, 5'-GATTCCATACCCAGGAAGG-3'.

ELISA and TransAM® NF-κB p65 assay

HBL1 cells were seeded at 300,000 cells/ml in RPMI 1640 plus 10% FBS, and then treated for 2 h with SHARPIN unstapled (20 μM) or SHARPIN-LTM (20 μM) peptides. The cells were stimulated with TNF-α (5 ng/ml) for 0, 5, 10, 30, or 60 min for determination of NF-κB p65 activation, and for 4 or 24 h for measurement of secreted IL-8. Cells were pelleted by centrifugation (250 × g, 5 min, 4°C). Supernatants and pellets were stored separately at -80°C until use. Secreted IL-8 in cell supernatants was measured using the Human IL-8 (CXCL8) Standard TMB ELISA Development kit (PEPROTECH). Pelleted cells were lysed and analyzed for NF-κB p65 activity using the TransAm NF-κB p65 ELISA kit.

SUPPLEMENTAL REFERENCES

Bernal, F., Tyler, A.F., Korsmeyer, S.J., Walensky, L.D., and Verdine, G.L. (2007). Reactivation of the p53 tumor suppressor pathway by a stapled p53 peptide. J. Am. Chem. Soc. *129*, 2456-2457.

Bochkov, Y.A., and Palmenberg, A.C. (2006). Translational efficiency of EMCV IRES in bicistronic vectors is dependent upon IRES sequence and gene location. Biotechniques *41*, 283-284, 286, 288 passim.

Cong, L., Ran, F.A., Cox, D., Lin, S., Barretto, R., Habib, N., Hsu, P.D., Wu, X., Jiang, W., Marraffini, L.A., *et al.* (2013). Multiplex genome engineering using CRISPR/Cas systems. Science *339*, 819-823.

Fujita, H., Rahighi, S., Akita, M., Kato, R., Sasaki, Y., Wakatsuki, S., and Iwai, K. (2014). Mechanism underlying IκB kinase activation mediated by the linear ubiquitin chain assembly complex. Mol. Cell. Biol. *34*, 1322-1335.

Kirisako, T., Kamei, K., Murata, S., Kato, M., Fukumoto, H., Kanie, M., Sano, S., Tokunaga, F., Tanaka, K., and Iwai, K. (2006). A ubiquitin ligase complex assembles linear polyubiquitin chains. EMBO J. *25*, 4877-4887.

Ran, F.A., Hsu, P.D., Wright, J., Agarwala, V., Scott, D.A., and Zhang, F. (2013). Genome engineering using the CRISPR-Cas9 system. Nat. Protoc. *8*, 2281-2308.

Sakamoto, H., Egashira, S., Saito, N., Kirisako, T., Miller, S., Sasaki, Y., Matsumoto, T., Shimonishi, M., Komatsu, T., Terai, T., *et al.* (2015). Gliotoxin suppresses NF-κB activation by selectively inhibiting linear ubiquitin chain assembly complex (LUBAC). ACS Chem. Biol. *10*, 675-681.

Shimizu, S., Fujita, H., Sasaki, Y., Tsuruyama, T., Fukuda, K., and Iwai, K. (2016). Differential Involvement of the Npl4 Zinc Finger Domains of SHARPIN and HOIL-1L in Linear Ubiquitin Chain Assembly Complex-Mediated Cell Death Protection. Mol. Cell. Biol. *36*, 1569-1583.

Tokunaga, F., Nakagawa, T., Nakahara, M., Saeki, Y., Taniguchi, M., Sakata, S., Tanaka, K., Nakano, H., and Iwai, K. (2011). SHARPIN is a component of the NF-κB-activating linear ubiquitin chain assembly complex. Nature *471*, 633-636.

Tokunaga, F., Sakata, S., Saeki, Y., Satomi, Y., Kirisako, T., Kamei, K., Nakagawa, T., Kato, M., Murata, S., Yamaoka, S., *et al.* (2009). Involvement of linear polyubiquitylation of NEMO in NF-kappaB activation. Nat. Cell Biol. *11*, 123-132.
